# SPARC: A Soft, Proprioceptive, Agile Robot for 3D Climbing and Exploration with Precise Trajectory Following

**DOI:** 10.1002/advs.202510382

**Published:** 2025-09-14

**Authors:** Weicheng Fan, Jiaqi Wang, Hongjian Zhang, Qichen Wu, Qihong Hu, Genliang Chen, Hao Wang, Xiaonan Huang

**Affiliations:** ^1^ State Key Laboratory of Mechanical Systems and Vibration, and Shanghai Key Laboratory of Digital Manufacturing for Thin‐Walled Structures Shanghai Jiao Tong University Shanghai 200240 China; ^2^ Hybrid Dynamic Robotics Lab, Robotics Department University of Michigan Ann Arbor MI 48109 USA; ^3^ Meta Robotics Institute Shanghai Jiao Tong University Shanghai 200240 China

**Keywords:** 3D navigation, autonomous trajectory following, origami soft actuator, proprioception, soft climbing robots

## Abstract

Soft robots, like their natural counterparts, are well‐suited for exploring 3D terrains due to their large deformation, impact resistance, and adaptive adhesion, derived from their inherent compliance. However, developing soft robots that can navigate both horizontal and vertical surfaces with seamless transitions and precise trajectory tracking remains a challenge. SPARC, a Soft, Proprioceptive, Agile Robot is presented for 3D climbing and exploration with accurate trajectory following. SPARC integrates three parallel 3D‐printed Kresling origami actuators with suction cups for 3D actuation and adhesion across diverse surfaces. Leveraging the contraction–twisting coupling of Kresling structures, SPARC enables real‐time proprioception through inverse kinematics modeling. A dual closed‐loop control strategy combined with efficient online path planning allows precise pose control using onboard angle sensors and global position tracking. Experiments demonstrate SPARC's ability to follow curved and sharply angled trajectories with high precision'achieving 0.5% accuracy on horizontal and 3% on vertical surfaces'while carrying payloads exceeding twice its weight (500 g) on vertical climbs. Moreover, connecting two SPARC modules enables smooth ground‐to‐wall transitions. The seamless integration of large‐magnitude 3D actuation, proprioceptive state sensing, and efficient online path planning makes SPARC a unique soft robot capable of precise trajectory following and navigation in 3D terrains.

## Graphical abstract

SPARC is a soft, proprioceptive, agile robot for 3D climbing, and exploration, integrating three parallel‐configured 3D‐printed Kresling origami actuators with embedded proprioception, a dual closed‐loop feedback control system, and pneumatic suction feet, enabling precise trajectory following in 3D, smooth transitioning from the ground to the wall, and payload carrying over twice its weight.

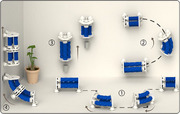

## 1 Introduction

To thrive in nature, many organisms have evolved sophisticated physical adaptations that enable them to navigate complex, 3D environments. For instance, earthworms (*Lumbricus terrestris*)^[^ ^1^ ^]^ and the Spicebush Swallowtail caterpillars (*Papilio troilus*)^[^ ^2^ ^]^ adeptly traverse soil, rocks, and branches using sequential muscular contractions and expansions, supported by mechanical anchoring mechanisms such as claspers and satae. Similarly, octopuses (*Octopus vulgaris*)^[^ ^3^ ^]^ use a grip‐and‐release technique, bending and twisting their arms and employing suction cups for effective movement across the sea floor and coral reefs. These 3D movements not only allow access to a wide range of feeding sites but also significantly enhance their ability to evade predators, showcasing the remarkable adaptability and resilience of life in navigating its environment.^[^ ^4^ ^]^ The 3D maneuverability observed in nature has spurred advances in robotics, leading to the development of robots with enhanced 3D movement and climbing capabilities. This progress has led to increased efficiency in areas such as construction and search and rescue, saving lives and reducing costs.^[^ ^5^, ^6^ ^]^ However, most of these robots are rigid, relying on complex actuation and adhesion mechanisms that result in bulky designs and reduced impact resistance.^[^ ^7^, ^8^, ^9^, ^10^ ^]^

The emerging field of soft robotics holds promise for 3D terrain exploration, attributed to its intrinsic compliance,^[^ ^11^, ^12^, ^13^ ^]^ adaptive adhesion capabilities^[^ ^14^, ^15^, ^16^, ^17^ ^]^ and formidable resistance to impacts.^[^ ^18^, ^19^, ^20^ ^]^ For example, Zhang et al. introduced a soft robot that emulates an inchworm's locomotion, equipped to crawl, climb, and seamlessly transition from ground to wall using suction cups.^[^ ^21^ ^]^ Similarly, Chen et al. showcased a versatile, lightweight, soft robot with dual electro‐adhesion pads, capable of navigating and transitioning across horizontal, vertical, and even overhead surfaces.^[^ ^22^ ^]^ However, in these studies, the robots' motion is limited to a vertical plane without the ability to turn. Recently, a few soft robots have demonstrated 3D turning capability using one or a pair of parallel modules. Nevertheless, these robots either lack payload capability on vertical planes^[^ ^13^, ^23^, ^24^, ^25^ ^]^ or sensory feedback, resulting in open‐loop systems for operational control.^[^ ^13^, ^19^, ^23^, ^24^, ^25^, ^26^, ^27^ ^]^ This limitation reduces their efficacy and practicality in real‐world scenarios. Additionally, many micro‐sized soft robots have been developed for climbing 3D surfaces utilizing magnetic‐driven^[^ ^28^, ^29^, ^30^, ^31^, ^32^ ^]^ and light‐driven^[^ ^33^, ^34^, ^35^ ^]^ methods. However, these approaches are predominantly effective in confined spaces (e.g., the human body) and unsuitable for open‐field applications. Consequently, to enable 3D climbing motion of soft robots on 3D surfaces in field environments, it is imperative to develop a highly integrated system with sufficient 3D actuation, strong adhesion, sensation, and feedback control.

Despite significant progress in the field of piece‐wise rigid robots for trajectory following and path planning,^[^ ^36^, ^37^, ^38^, ^39^, ^40^ ^]^ few soft robots can achieve these due to their complex material properties,^[^ ^41^ ^]^ lack of precise control mechanisms,^[^ ^42^ ^]^ and challenges in accurate modeling and feedback integration.^[^ ^43^ ^]^ External sensor‐based visual servoing is a dominant control strategy among the tracking methods. By integrating PID (proportional‐integral‐derivative) control,^[^ ^44^, ^45^ ^]^ dynamic models,^[^ ^46^, ^47^ ^]^ and fusion perception methods,^[^ ^48^ ^]^ some soft mobile robots have achieved autonomous motion capabilities for trajectory tracking and obstacle avoidance. Oyuna et al. introduced a visual PID control method to enable preliminary autonomous locomotion in soft crawling robots. However, this approach fails to achieve navigation through sharp‐angle turns and is constrained to overly smooth trajectories.^[^ ^45^ ^]^ By leveraging the 2D discrete elastic rods simulation, Huang et al. developed a closed‐loop motion planning system that enables an untethered swimming soft robot to track trajectory in low‐resistance environments. Nevertheless, a single visual feedback is insufficient to detect the motion state of the robot's actuators, resulting in notable errors (approximately 10 cm).^[^ ^46^ ^]^ To address these issues, Lu et al. proposed a fusion perception method that combines offboard cameras and onboard length sensors to simultaneously capture the overall pose and individual actuator lengths, achieving real‐time precise pose perception of the tensegrity robot. However, the point cloud registration methods employed in this approach (e.g., point pair features, iterative closest point) struggle to balance efficiency and accuracy.^[^ ^48^ ^]^ The acquisition of high‐precision, high‐resolution point clouds required for accurate registration results significantly slows down system performance and increases operational costs. While a few soft mobile robots with feedback control have been developed for horizontal movement, most cannot navigate vertical surfaces or transition between planes due to insufficient compliance and anchoring mechanisms to counteract gravity.^[^ ^23^, ^31^, ^49^, ^50^, ^51^, ^52^, ^53^, ^54^ ^]^ Consequently, to the best of our knowledge, no soft robots can currently perform precise motion planning in 3D terrain.

**Figure 1:**
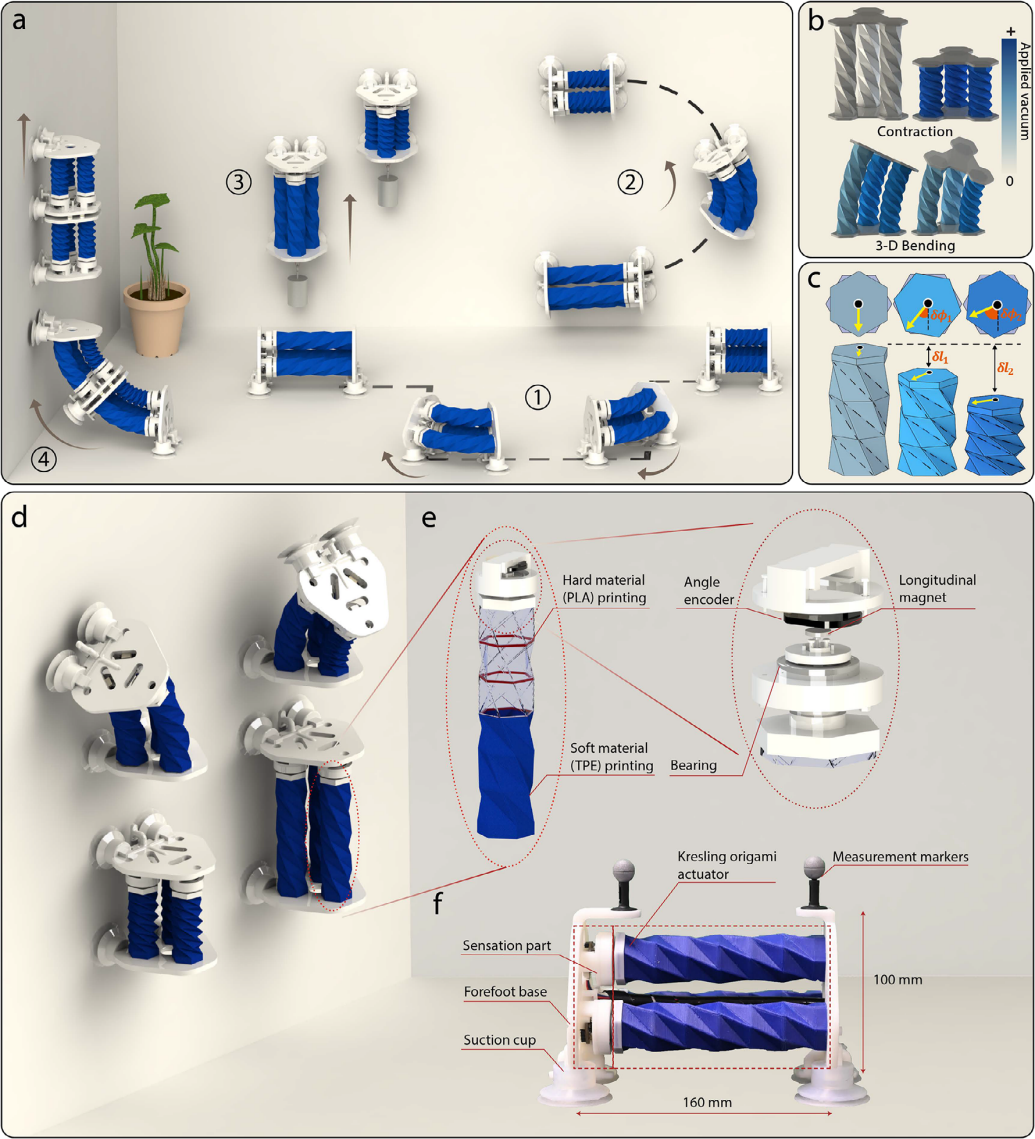
Versatile 3D locomotion of SPARC in complex environments. a) Overview of SPARC's locomotion capabilities, demonstrating its ability to follow various trajectories with high precision on both horizontal (①) and vertical surfaces (②), and to carry loads exceeding twice its weight (500 g) on vertical surfaces (③). The series connection of two modules facilitates ground‐to‐wall transitions (④). b) Schematic of SPARC's 3D bending motion, illustrating how applying different vacuum levels to the three actuators enables either pure contraction or omnidirectional bending. c) Schematic of the unique contraction‐twisting coupling features of Kresling origami actuators, showing a one‐to‐one mapping between contraction distance (δ*l*) and twisting angle (δϕ), which enables proprioception of the actuator's length. d) Motion modes of SPARC, including forward locomotion, turning, and detachment from surfaces. e) An origami linkage module comprising a 3D‐printed soft TPE body, rigid inner PLA rings, diameter‐magnetized magnets, angle sensors, and bearings. f) Core components of SPARC, featuring vacuum‐driven 3D‐printed Kresling origami actuators for motion, sensors to measure twisting angles, suction cups for adhesion, and measurement markers for global tracking.

To address these challenges in soft robotics, we present SPARC, a **s**oft, **p**roprioceptive, **a**gile **r**obot designed for 3D **c**limbing and exploration, capable of dynamically navigating complex trajectories on both ground and vertical surfaces (**Figure** **1**). It achieves seamless transitions between these orientations and can carry payloads (500 g) exceeding twice its own weight (210 g) on vertical planes (Figure 1a). The high‐magnitude contraction (60%) and bending (50 degrees) in 3D, along with its high load‐carrying capability, are attributed to the incorporation of three pneumatic Kresling origami actuators configured in parallel (Figure 1b). Vacuum‐driven soft actuators with Kresling origami patterns are known for their high contraction ratio and high force density.^[^ ^55^, ^56^, ^57^ ^]^ Here, we innovatively leverage the unique contraction‐twisting coupling characteristic inherent to Kresling origami to enable real‐time proprioception and state reconstruction (Figure 1c), which remains robust to payload variation. To facilitate accurate trajectory following, we incorporate a dual closed‐loop control system within the gait controller. This system employs angle encoders for precise pose management alongside motion capture technologies to adjust the robot's global positioning. For path planning, we have implemented a pure pursuit algorithm that can accurately track curved paths while adeptly navigating sharp turns, ensuring the robot's effective maneuverability and agility across complex terrains. The tight integration of 3D actuation, innovative proprioception capabilities, effective kinematics modeling, robust control, and efficient planning strategies makes SPARC, to our knowledge, the first soft robotic system capable of precise motion planning in 3D terrain.

Overall, the contributions of our work can be summarized as follows:

1) SPARC, a soft, proprioceptive, agile robot for 3D climbing, and exploration, integrating three parallel‐configured 3D‐printed Kresling origami actuators with embedded proprioception, a dual closed‐loop feedback control system, and pneumatic suction feet, enabling precise trajectory following in 3D, smooth transitioning from the ground to the wall, and payload carrying over twice its weight.
2) A compact, high‐fidelity sensing scheme leveraging the embodied mechanical intelligence of the Kresling actuator (i.e., contraction‐twist coupling) for real‐time proprioception and state reconstruction of the robot.
3) A robust dual closed‐loop control and path‐planning system combining local actuator control and global positional feedback for precise, agile trajectory following in 3D.

## 2 Results

### 2.1 Robot Design and Fabrication

To create a soft robot capable of precise locomotion in 3D terrain, carrying heavy payloads, and smoothly transitioning from horizontal to vertical planes, the robot must achieve high‐magnitude and high‐force contraction, 3D bending, as well as strong adhesion. To this end, we employed a Kresling origami design^[^ ^58^ ^]^ and developed a pneumatic origami soft actuator. This actuator boasts an exceptional contraction ratio of 60% at ‐80 kPa, an actuation force of 3 kg (Figure S2, Supporting Information), and a service life of over 20,000 cycles (Figure S3, Supporting Information). These capabilities are attributed to the high unfolding ratio of the origami patterns, vacuum‐based actuation, and high‐quality 3D printing, respectively. Additionally, we innovatively leveraged the embodied mechanical intelligence ‐ specifically, the intrinsic contraction‐twisting coupling motion characteristic of Kresling origami patterns ‐ to enable the actuator to self‐sense and dynamically adjust its length in real‐time. Three linear Kresling actuators are configured in parallel to facilitate both contraction and 3D bending (Figure 1d). Each actuator consists of six chirality‐preserving (i.e., the direction of twisting remains consistent after compression) Kresling basic units connected in series. Rigid support rings are inserted between each pair of units to prevent deformation of the hexagonal cross‐section, thus avoiding radial contraction and ensuring purely linear motion. While many studies utilizing Kresling origami chambers as linear actuators focus on suppressing undesired torsional motion at the distal end, our design actively exploits this behavior: the chamber terminus is kinematically coupled to the bearing's inner ring via 3D‐printed connectors secured with bolted fasteners, while the outer ring is rigidly mounted to the forefoot base (Figure S4, Supporting Information), thereby decoupling the actuator's twisting deformation from the overall structure and eliminating its influence on axial contraction. Furthermore, by attaching a miniature angle encoder to the bearing at one end of each actuator, we enable pure axial contraction while simultaneously measuring the twisting angle and converting it to actuator length, integrating this into an actuation‐sensing‐structure system (Figure 1e). For adhesion, SPARC utilizes four silicone suction cups that serve as feet, allowing the robot to adhere to and traverse 3D terrain. To mitigate the risk of distortion in the silicone suction cup, we use a dual suction cup configuration for each foot, ensuring stable position and orientation near the foot during vacuum adhesion. Additionally, two measurement markers are installed on the robot to provide self‐positioning feedback to the control systems (Figure 1f).

To ensure uniform contraction within the actuator and performance consistency among actuators, as well as reducing labor costs in fabrication, the origami actuator is 3D printed using a combination of soft and hard materials (Figure S5, Supporting Information, see “Fabrication of the origami chambers” in Experimental Section, with Kresling dimensions shown in Figure S8 and Table S1, Figure S5, Supporting Information). We use TPE (83A, Thermoplastic Elastomer) as the soft material for the actuator. This material is similar to TPU (95A, Thermoplastic Polyurethanes) but has a lower Shore hardness and better fluidity, resulting in TPE‐manufactured chambers with fewer wall imperfections and enhancing the actuator's airtightness. The remaining rigid components are 3D printed using PLA material, and the vacuum suction cups are prepared from silicone rubber (45A, Kairuisi).

### 2.2 Proprioception Modeling and Validation

To employ the proprioception feature of the Kresling origami actuators in locomotion control, finite element analysis (FEA) is applied to establish the mapping between the actuator's length and twisting angle. Additionally, we conduct uniaxial tensile tests on TPE samples after 3D printing to account for possible alterations in material properties due to the 3D printing process (see “Uniaxial tensile test” in Supporting Information).

**Figure 2:**
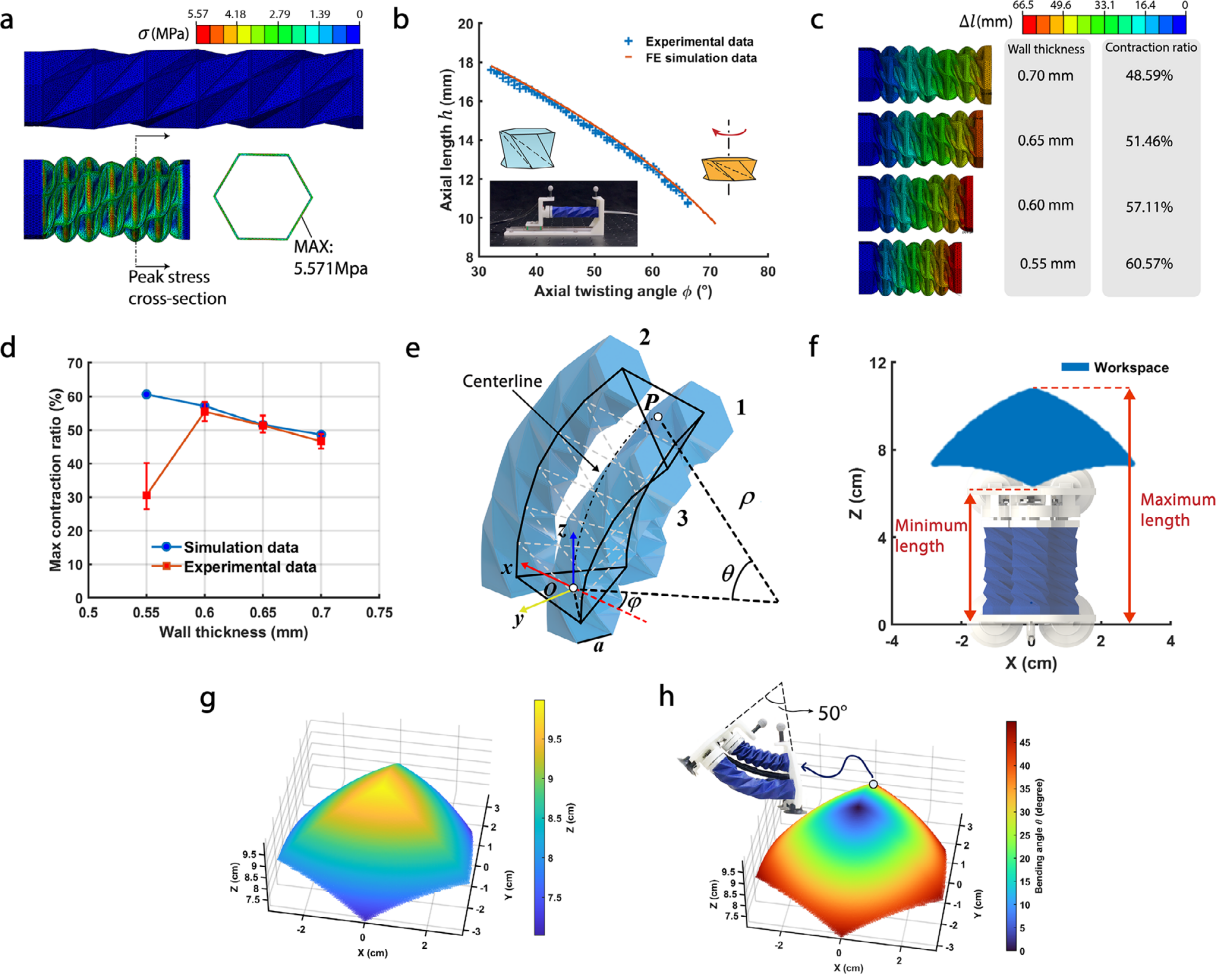
Simulation of Kresling origami actuators and kinematics modeling of SPARC. a) Finite element analysis illustrating the contraction process of the actuator, with peak stress occurring at the center of the side of the hexagonal cross‐section (wall thickness of 0.6 mm, under ‐60 kPa). b) Comparison of simulated and experimental data for the relationship between axial length and twisting angle of a single‐section origami actuator. c) Simulation revealing the relationship between maximum contraction ratio and wall thickness (under ‐60 kPa). d) Comparison of simulated and experimental data for the relationships between maximum contraction ratio and wall thickness (under ‐60 kPa). e) Inverse kinematic model of SPARC. f) 2D workspace of SPARC. g) 3D workspace of SPARC. h) Bending angles within SPARC's workspace, with the maximum bending angle reaching approximately 50 degrees.

Based on the material properties obtained from uniaxial tensile tests, we conduct finite element analysis on the folding process of the Kresling origami chamber using computer‐aided engineering software (see “Numerical simulation” in Experimental Section). **Figure** **2**a displays the results of the finite element analysis, revealing the relationship between the length of the actuator and its self‐twisting angle. Additionally, it shows that the maximum stress is concentrated at the outer edge of the non‐collapsible hexagonal folds, which is consistent with the fatigue damage observed in the experiments. By using our fitting methods^[^ ^55^ ^]^ (see “Geometrical analysis of Kresling origami” in Supporting Information), a self‐perception model is derived:

(1) $$\begin{array}{cc} l & =f(\Phi ) \end{array}$$

here, *l* is the length of the actuator, Φ is the axial twisting angle, and *f* is demonstrated in Equation (S5) (Supporting Information). Three repeated physical experiments with different actuators are conducted to validate the model. Figure 2b illustrates the experimental setup and the results of experimental validation. The RMSE of the experimental data relative to the model is 0.4438 mm, indicating that the finite element modeling approach, validated by experiments, is highly reliable. The FEA and experimental results are available in Movie S1 (Supporting Information). For Kresling origami chambers under bending conditions, experimental investigations in our previous work^[^ ^59^ ^]^ demonstrated that the contraction behavior remains consistent with Equation (1) within acceptable tolerances.

### 2.3 Parameter Sweep for Origami Actuators

The wall thickness plays a crucial role in the functionality of pneumatic actuators within soft robotic systems. Excessive thickness leads to increased stiffness in the actuators, diminishing their contraction ratio and, consequently, restricting the robot's operational workspace. On the other hand, employing FDM 3D printing techniques with too thin layers may induce cracks, jeopardizing the actuator's integrity by causing gas leaks, which similarly constrains the robot's workspace, as well as load‐bearing capability. Here, we determine this optimal thickness by combining FEA simulation and experiments. Given the input pressure as ‐60 kPa, we have observed that this ratio varies as the thickness parameters of the actuator are modified. Figure 2c,d illustrate the influence of the wall thickness of the origami chambers on the maximum contraction ratio. It is evident that a smaller wall thickness leads to a higher maximum contraction ratio. However, physical experiments do not align with this observation. The actuator becomes susceptible to cracking and damage during experiments when the wall thickness falls below a certain threshold (0.6 mm in this particular design). In the experiment, cracking occurs at 0.55 mm wall thickness, compromising the chamber's integrity, and causing air leakage that prevents the vacuum‐generated negative pressure from being fully transmitted to the inner walls of the chamber. Consequently, the internal pressure fails to attain the preset value used in our FEA simulations, leading to diminished driving force and reduced deformation. To avoid failure, a wall thickness of 0.6 mm is selected for the origami chambers, ensuring structural integrity without failure. If more advanced manufacturing methods are available, we can further reduce the thickness of the chamber wall while preventing rupture or leakage under folding and vacuum loading. Details of the wall thickness optimization process by simulation are provided in the section titled “Wall Thickness Optimization of the Kresling Origami Actuator by Simulation” in the Supporting Information.

### 2.4 Kinematic Model

Unlike those with rigid links, robots made from soft actuators are challenging to model primarily due to their intrinsic, continuous, complex, and highly compliant deformation.^[^ ^60^ ^]^ To reveal the mapping from the actuator space to the robots' configuration space for controlling robots effectively, the constant curvature (CC) assumption is widely used for its simplicity and effectiveness^[^ ^61^, ^62^, ^63^, ^64^ ^]^ For multisection continuum/soft robots, each CC section can be linked together to form a piecewise constant curvature (PCC) model.^[^ ^65^ ^]^ In this work, we use the PCC model for inverse kinematic modeling of SPARC. The model relates the position of the forefoot to the lengths of the three actuators when the hindfoot suction cup is fixed and the forefoot suction cup is released:

(2) $$\begin{array}{cc} l_{i} & =2n\sin\frac{\theta }{2n}\left[\rho -a\cos\left(\frac{2\pi }{3}\left(i-1\right)+\frac{\pi }{2}-\varphi \right)\right] \end{array}$$

where

(3) $$\begin{array}{cc} & \varphi =\tan^{-1}\left(\frac{y}{x}\right)\\ & \rho =\frac{x^{2}+y^{2}+z^{2}}{2\sqrt{x^{2}+y^{2}}}\\ & \theta =\left\{\begin{array}{cc} \cos^{-1}\left(1-\frac{\sqrt{x^{2}+y^{2}}}{\rho }\right), & \text{if}\;z>0\\ 2\pi -\cos^{-1}\left(1-\frac{\sqrt{x^{2}+y^{2}}}{\rho }\right), & \text{if}\;z\leq 0 \end{array}\right. \end{array}$$

here, *l*_*i*_ (*i* = 1, 2, 3) represents the lengths of the three actuators, (*x*, *y*, *z*) denotes the position of the endpoint P, and *a* reflects the distance between the centers of the chambers' bases. The configuration space of SPARC can be defined by arc parameters, including the radius ρ, bending angle θ, and the rotated angle in the *o*‐*xy* plane φ of the centerline. Figure 2e provides details of the model.

Based on the inverse kinematic model, we determined the one‐step motion space of SPARC utilizing the Monte Carlo method^[^ ^66^ ^]^ Figure 2f and g display the 2D and 3D views of the workspace, respectively. The results indicate that its maximum forward step length is 30 mm, and the workspace configuration resembles that of a cone, characterized by a bottom diameter of 60 mm and a height of 30 mm. Furthermore, Figure 2h illustrates the bending angles of SPARC within its 3D workspace. The results indicate a maximum bending angle of approximately 50 degrees, aligning with experimental findings.

### 2.5 Gait Design and Trajectory Following

**Figure 3:**
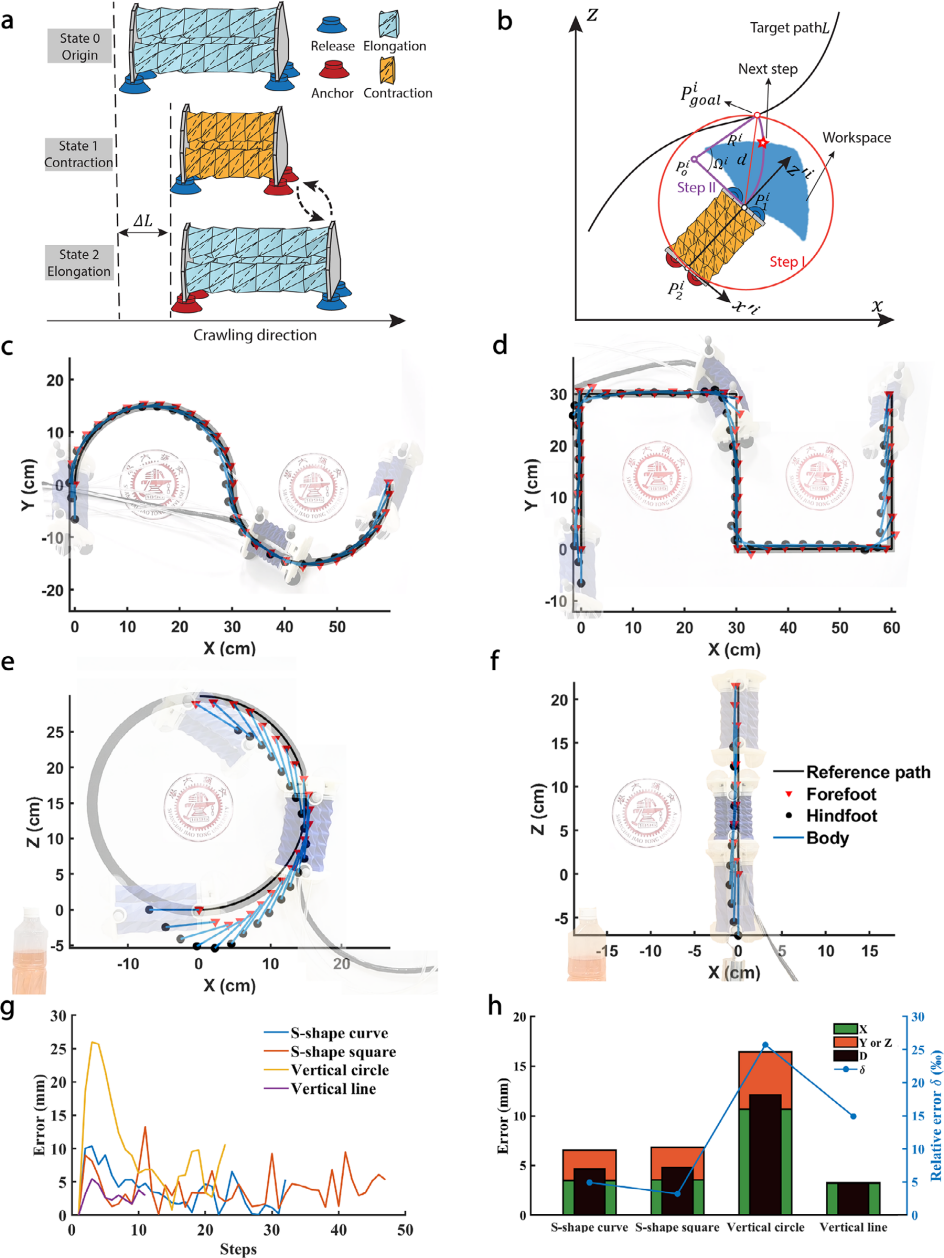
Path following controller and experimental validation. a) Crawling motion of SPARC which includes a forward gait cycle sequence of anchoring the forefoot, contracting chambers, anchoring the hindfoot, and extending chambers. b) Schematic of the path tracking algorithm, with the red star indicating SPARC's next step. Experimental validation: c) An S‐shape curve on the horizontal plane (curve radius = 15 cm), d) an S‐shape square on the horizontal plane (square side length = 30 cm), e) a semicircle on the vertical plane (curve radius = 15 cm), and f) a straight line on the vertical plane carrying a 500 g load. g) Translation errors between the actual and theoretical positions at each step. **h**, Average errors across the entire trajectory.

Earthworms alternate between contracting and relaxing muscles, aided by bristles that adjust to propel or minimize resistance, enabling efficient soil movement^[^ ^67^, ^68^ ^]^ Inspired by their movement pattern, we designed the crawling gait of SPARC, as shown in **Figure** **3**a. Starting with the initial state 0, a forward gait cycle consists of a sequence of forefoot anchored, chambers contraction, hindfoot anchored, and chambers extension. During step 1, negative pressure is applied to the front suction cups to secure them in place, followed by the contraction of three Kresling actuators while the rear foot is free (state 1). Subsequently, in step 2, with the rear foot held in place, the front foot is detached, and the negative pressure on the actuators is released, allowing them to return to their original length (state 2). These two steps constitute a cycle to propel SPARC forward by a distance of Δ*L* (30 mm). The continuous motion of SPARC is comprised of multiple gait cycles. To achieve reliable and successful adhesion on smooth surfaces, we employed a strategy in our experiments that involved applying a pre‐loaded downward force to the suction cups, thereby increasing the contact area between the suction cup margins and the surface to ensure reliable sealing (see the section titled “Reliable adhesion based on preloaded‐force strategy” in the Supporting Information for details). With this gait pattern, SPARC achieves not only crawling locomotion but also demonstrates climbing capability across varied terrains and under different payload conditions (see “Terrain Adaptability and Load Carrying Experiment” in Supporting Information for details on the calculation method).

**Figure 4:**
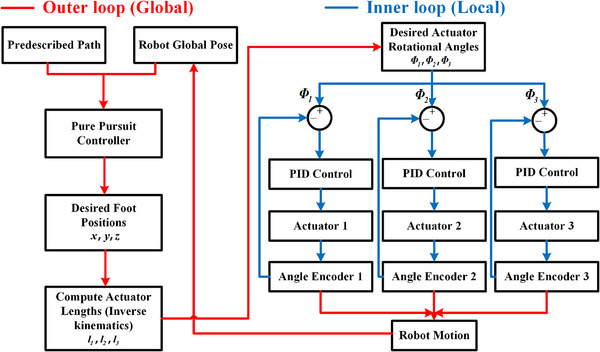
Dual‐loop control system architecture diagram, comprising: 1) a system‐level outer loop control for trajectory following, based on robot global positional feedback (indicated by red lines) and 2) an actuator‐level inner loop control utilizing local end‐effector angular encoder feedback (indicated by blue lines).

By leveraging this gait and the pure pursuit controller,^[^ ^69^ ^]^ we have developed a tracking control algorithm for SPARC (Algorithm 1). After inputting the prescribed trajectory L and the initial positions of the fore and hind legs $\left(P_{1}^{1},P_{2}^{1}\right)$, SPARC approaches the endpoint $P_{\text{final}}$ along the trajectory L through a gait control algorithm in each step (Figure 3b, see “Gait control algorithm” in Experimental Section and “Pure pursuit controller” in the Supporting Information). This algorithmic architecture implements a dual‐loop closed‐loop feedback control system (**Figure** **4**), comprising: 1) a system‐level trajectory tracking outer loop control based on robot global positional feedback; 2) an actuator‐level inner loop control utilizing local end‐effector angular encoder feedback. In the outer loop, the optical sensor captures the robot's current pose, which is combined with the prescribed path and input into the pure pursuit controller. The controller calculates the coordinates of the next foothold. Subsequently, the inverse kinematics (Equation (2)) determine the required lengths of the three actuators and the length‐to‐angle mapping relationship (Equation (1)) derives the target rotation angles for each actuator. These angle values are then transmitted to the inner loop. Within the inner loop, angle encoders measure the actual rotation angles of the actuators as feedback signals, with the negative pressure input serving as the control variable. Using PID controllers, the three actuators are driven to achieve their respective target rotation angles, thereby enabling the robot to complete the desired motion for each step. Equipped with this architecture, SPARC achieves high‐precision trajectory tracking.

Algorithm 1 **Tracking control algorithm.**

**Table:** 

	**Procedure** (L, $P_{1}^{1},P_{2}^{1}$).
1:	*i* = 1
2:	$P_{\text{final}}\leftarrow$ Endpoint of L
3:	*e* ← $\left\|\vec{P_{1}^{1}P_{\text{final}}}\right\|$
4:	**while** *e* ⩾ ε (the precision criterion) **do**
5:	$(P_{1}^{i+1},P_{2}^{i+1})\leftarrow$ $F_{1}(L$, $P_{1}^{i},P_{2}^{i})$
6:	*i* = *i* + 1
7:	*e* ← $\left\|\vec{P_{1}^{i}P_{\text{final}}}\right\|$
8:	**end while**

### 2.6 Trajectory Following Experiment

To validate SPARC's trajectory‐following capability on both horizontal and vertical planes, with and without carrying a payload (500g), we design four distinct trajectories: a S‐shape curved path and a sharply angled path on the horizontal plane, along with straight and curved paths on the vertical plane (see “Trajectory design” in Supporting Information).

Four predetermined trajectory‐following tests are conducted using the SPARC system following thorough experimental preparation in Figure S6 (Supporting Information) (see “Experimental settings and setups” in Experimental Section for details). The trajectory following results for the four designed trajectories are demonstrated in Figure 3c–f with corresponding Movies [Link], [Link], [Link], [Link] (Supporting Information). Figure 3c and d show that SPARC successfully follows the predefined trajectory on the horizontal plane. Due to the algorithm's focus on the forefoot's trajectory tracking, a slight deviation is observed in the hindfoot during square turns. During the arc trajectory tracking on the vertical plane, SPARC initially experiences a downward deviation due to gravity. However, the dual closed‐loop algorithm progressively corrects this deviation in the latter half, as illustrated in Figure 3e. Furthermore, Figure 3f showcases SPARC's ability to ascend vertically on a wall while carrying a 500g load, approximately twice its weight, following a straight upward trajectory. Figure 3g,h summarize these results, indicating SPARC's high motion accuracy on the ground, with a relative tracking error of approximately 0.5% (see “Trajectory error calculation” in Supporting Information for details on the calculation method). Even in the experiment involving tracking curved trajectories on a vertical wall, SPARC exhibits only a trajectory deviation within 3%. These results underscore SPARC's precise and autonomous locomotion capability both on horizontal and vertical surfaces. Moreover, the tracking errors in both directions of SPARC are comparable on both S‐shape curve and square trajectories, demonstrating the excellent stability and robustness of its control system. During the vertical line following, errors are all manifested in the X direction since the nearest waypoint on the trajectory to the forefoot (i.e., the orthogonal point) consistently shares the same Z value as the forefoot.

### 2.7 Serial Configuration of Two SPARC Modules for Enhanced Terrain Adaptability

**Figure 5:**
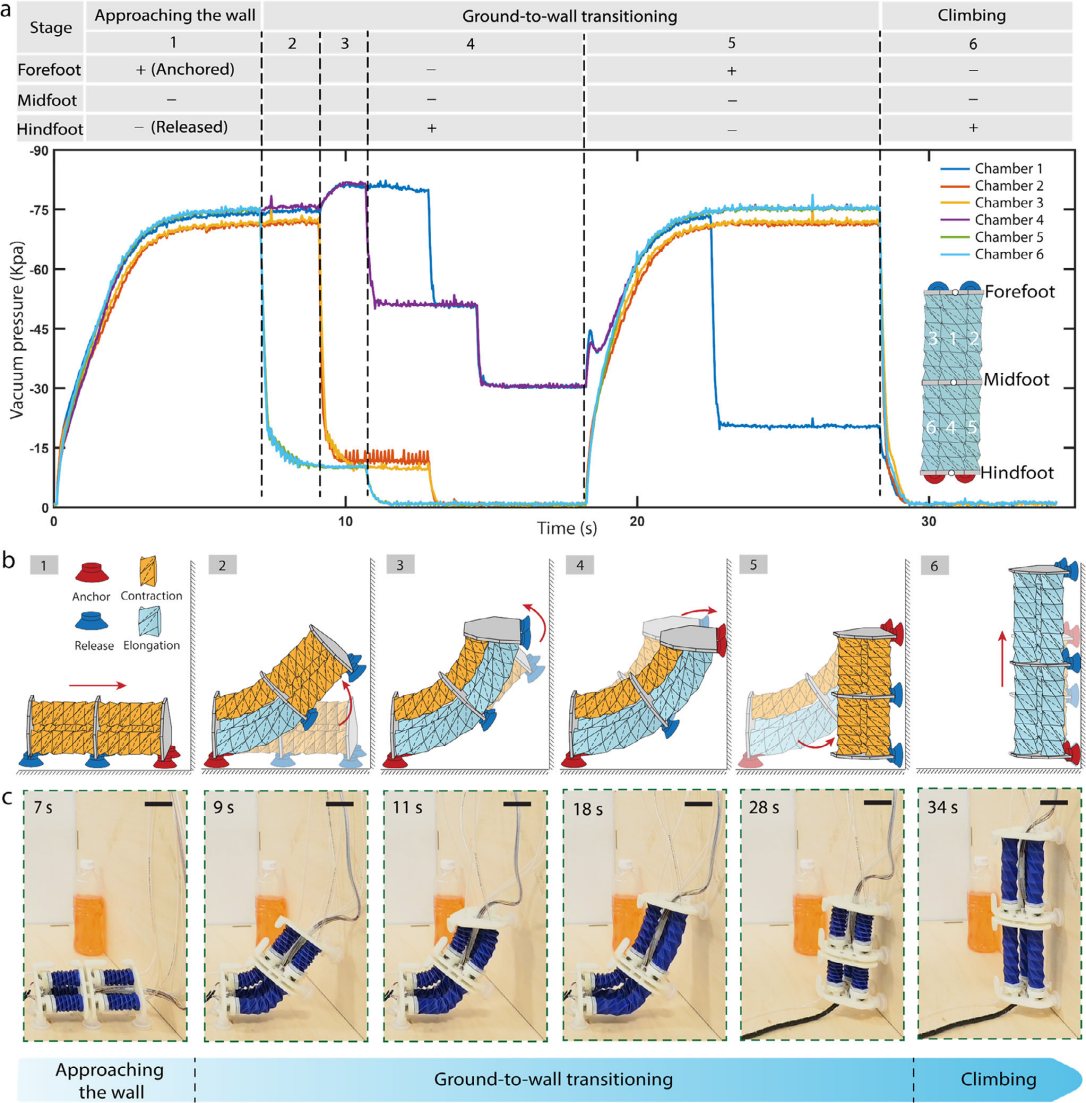
Locomotion strategy and demonstration of ground‐to‐wall transition with the serially configured SPARCs. a) Monitored sequence showing the status of suction cups and the six‐channel input vacuum during the ground‐to‐wall transition, which encompasses approaching the wall, transitioning from ground to wall, and climbing. b) Schematic illustration and locomotion strategy detailing the transition process (from “1” to “6”) for serially configured SPARC between two orthogonal walls: 1) approach the wall, 2) lift the midfoot and forefoot, 3) elevate the forefoot to the highest position, 4) adjust the forefoot to identify the appropriate landing point, 5) anchor the forefoot on the wall and contract the whole body, 6) anchor the hindfoot and ascend. c) A sequence of frames showing the serially configured SPARCs transitioning from ground to wall. (Scale bars represent 5 cm).

Connecting and reconfiguring multiple identical robotic modules can significantly improve the robot's range of motion and enhance its adaptability.^[^ ^70^, ^71^, ^72^ ^]^ By connecting two SPARC modules in series, we create a serial configuration of SPARC, enabling a theoretical maximum bending angle of 100 degrees. This configuration allows the robot to smoothly transition from ground to walls that are orthogonal to each other (i.e., 90 degrees) (**Figure** **5** and Movie S6, Supporting Information). Additionally, the serial connection enables a maximum step length twice that of a single segment (Figure S7, Movie S6, Supporting Information).

The ground‐to‐wall transition motion is initiated as the robot's forefoot approaches the wall (within 2 cm). At this point, the robot contracts all origami actuators to their minimum lengths, securing the hindfoot in preparation for vertical bending (refer to Figure 5b,c, step 1).

To ensure a seamless transition with the forefoot being accurately placed against the wall, the transition is executed in four coordinated steps. Initially, the lower origami actuators on the rear SPARC module (chambers 5 and 6, as shown in Figure 5a) are depressurized to extend, causing the rear section to bend upwards (Figure 5b,c, step 2). Subsequently, the lower actuators on the front module (chambers 2 and 3) extend through depressurization, arching the entire SPARC upwards for approximately 100 degrees (Figure 5b,c, step 3). The next step involves fine‐tuning the upper origami actuators on both front and rear modules via manual control to press the forefoot against the wall, followed by activating the forefoot's suction cup for stable attachment (Figure 5b,c, step 4). The transition culminates with all actuators retracting to their minimum lengths, securing the robot against the wall, and positioning it for the climbing phase (Figure 5b,c, step 5).

The climbing motion closely mirrors the ground crawling movement, characterized by a forward gait cycle that includes anchoring the hindfoot, extending all actuators, anchoring the forefoot, and then contracting all actuators (Figure 5b, step 6). A minor adaptation for wall climbing involves utilizing a reduced negative pressure in chamber one to facilitate a smoother transition, avoiding direct extension to full length. This gait adjustment allows SPARC's head to tilt and extend upwards initially, then descend, effectively minimizing collision risks with the wall during ascent.

## 3 Discussion

In this paper, we introduced SPARC, a soft, proprioceptive, agile robot for 3D climbing and exploration that excels in precise trajectory following. The combination of modular design, innovative proprioception capabilities, accurate kinematics modeling, robust control, and efficient planning strategies enables SPARC to navigate and maneuver through complex 3D terrains with remarkable precision and agility. SPARC incorporates three parallel 3D‐printed Kresling origami actuators, which facilitate a wide range of motion in 3D, high load‐carrying capability, and real‐time state sensing, thanks to their high contraction ratio, high force density, and embodied mechanical intelligence (i.e., correlation between twisting and contraction), respectively. To quantitatively summarize SPARC's locomotion performance, it is capable of i) accurately tracking curved paths and adeptly navigating sharp turns with accuracy within 0.5%; ii) following semicircle paths on vertical planes with accuracy within 3%; iii) carrying loads of 500 g, more than twice its weight (210 g), on vertical planes; and iv) achieving seamless ground‐to‐wall transition with two SPARC modules connected in series.

Compared with previous demonstrations of soft climbing robots (**Table** **1**), SPARC stands out in its ability to navigate both horizontal and vertical planes with smooth transitions, perform turning and symmetric forward‐backward movements on both planes, and carry loads twice its body weight, excelling in locomotion and load‐carrying ability. Leveraging a dual closed‐loop feedback control system, SPARC distinguishes itself as the first soft mobile robot capable of high‐precision trajectory tracking in 3D terrains. Unlike previous soft robots, which have an (estimated) trajectory tracking error exceeding 10%,^[^ ^44^, ^45^, ^46^, ^47^ ^]^ SPARC achieves a tracking error within 0.5%, even on trajectory with sharp turns. Moreover, no existing soft robots have demonstrated high‐precision trajectory tracking on vertical walls. In contrast, SPARC is capable of following a hemisphere curve on a vertical plane with a trajectory tracking error within 3%.

**Table 1:** Locomotion performance metrics of SPARC and other representative soft mobile robots.

Ref.	Design concept						Mobile ability				Autonomy		
	Adhesion	Actuation	Mass (g)	BL* (mm)	Modular	Proprioceptive actuators	Turning	Wall Climbing	Load/weight (on the wall)	Wall transition	Motion control (%)	Horizontal plane (%)	Wall (%)
SPARC	Pneumatic	Pneumatic	210	160	Yes	Yes	Yes	Yes	238%	Yes	Close‐loop	0.5%	3
Other representative soft mobile robots in field environment													
^[^ ^11^ ^]^	Frictional contact	Pneumatic	N/A*	135.7	/*	/	/	/	/	/	Open‐loop	/	/
^[^ ^12^ ^]^	Corkscrew gripper	Motor‐tendon	120.64	235	/	/	/	Yes	N/A	Yes	Open‐loop	/	/
^[^ ^13^ ^]^	Pneumatic	Pneumatic	N/A	N/A	Yes	/	Yes	Yes	N/A	Yes	Open‐loop	/	/
^[^ ^15^ ^]^	Pneumatic	Pneumatic	40	180	/	/	/	Yes	500%	/	Open‐loop	/	/
^[^ ^16^ ^]^	Electrically activated hydrogels	Motor	113/175/180	N/A	/	/	/	Yes	N/A	/	Open‐loop	/	/
^[^ ^19^ ^]^	Electromagnetic/Pneumatic	Electrothermal (SMA)	73.6/35.8	129/128	Yes	/	Yes	Yes	312%/1005%	Yes	Open‐loop	/	/
^[^ ^21^ ^]^	Pneumatic	Pneumatic	33	190	/	/	/	Yes	60%	Yes	Open‐loop	/	/
^[^ ^22^ ^]^	Electroadhesion	Pneumatic	2.57	48	/	/	/	Yes	N/A	Yes	Open‐loop	/	/
^[^ ^23^ ^]^	Magnetic	Piezoelectric	1.48	45	/	/	Yes	Yes	N/A	/	Open‐loop	/	/
^[^ ^25^ ^]^	Asymmetric friction	Piezoelectric	0.63	60	/	/	/	Yes	N/A	/	Open‐loop	/	/
^[^ ^24^ ^]^	Electroadhesion	Electrostatic	0.2‐3	6‐90	/	/	/	Yes	N/A	Yes	Open‐loop	/	/
^[^ ^26^ ^]^	Pneumatic	Motor‐flexible tubes	N/A	182	/	Yes	Yes	Yes	N/A	Yes	Open‐loop	/	/
^[^ ^27^ ^]^	Electroadhesion	Electrostatic (DE)	43.4	135	/	/	Yes	Yes	23%	/	Open‐loop	/	/
^[^ ^44^ ^]^	Electromagnetic	Electromagnetic	450	250	Yes	/	Yes	/	/	/	Close‐loop	∼15*	/
^[^ ^45^ ^]^	Sliding ratchet	Motor	165.3	155	Yes	Yes	Yes	/	/	/	Close‐loop	∼10	/
^[^ ^46^ ^]^	/	Electrothermal (SMA)	N/A	N/A	/	/	Yes	/	/	/	Close‐loop	∼30	/
^[^ ^47^ ^]^	Wheel	Electrothermal	N/A	90	/	/	Yes	/	/	/	Close‐loop	∼20	/
^[^ ^48^ ^]^	/	Motor‐Tensegrity	N/A	N/A	/	Yes	Yes	/	/	/	Open‐loop	/	/
^[^ ^49^ ^]^	Electroadhesion	Piezoelectric	0.065	30	/	/	Yes	/	/	/	Open‐loop	∼10	/
^[^ ^50^ ^]^	Ratchet	Pneumatic	48	152	/	/	Yes	/	/	/	Open‐loop	∼5	/
^[^ ^51^ ^]^	/	Magnetic	N/A	3.6	/	/	Yes	/	/	/	Open‐loop	∼5	/
^[^ ^52^ ^]^	Asymmetric friction	Electrostatic (DE)	4.8	87	/	/	Yes	/	/	/	Open‐loop	N/A	/
^[^ ^53^ ^]^	Asymmetric friction	Magnetic	0.95	28	Yes	/	Yes	/	/	/	Open‐loop	/	/
^[^ ^54^ ^]^	Caterpillar feet	Pneumatic	N/A	154	Yes	/	Yes	/	/	/	Open‐loop	/	/
Representative soft mobile robots in confined space													
^[^ ^28^ ^]^	Dry adhesion	Magnetic	0.003	3.7	/	/	Yes	Yes	2000%	Yes	Open‐loop	/	/
^[^ ^29^ ^]^	/	Magnetic	N/A	6.8	/	/	Yes	/	/	/	Open‐loop	/	/
^[^ ^30^ ^]^	Dry adhesion	Magnetic	0.039	17	/	/	/	/	/	/	Open‐loop	/	/
^[^ ^31^ ^]^	/	Magnetic	N/A	40	Yes	/	Yes	/	/	/	Open‐loop	/	/
^[^ ^32^ ^]^	Dry adhesion	Magnetic	N/A	4/8/12	/	/	Yes	Yes	/	Yes	Open‐loop	/	/
^[^ ^33^ ^]^	/	Light	N/A	21	/	/	/	/	/	/	Open‐loop	/	/
^[^ ^34^ ^]^	/	Light	N/A	13	/	/	/	/	/	/	Open‐loop	/	/

* “BL”= “Body length”; “N/A”= “Not available”; “/”= “Can't achieve”; “∼”= “Estimated by authors”

To deploy SPARC in the field for real‐world applications, such as pipeline inspection, hull monitoring, surveillance, and reconnaissance, we plan to “cut the cord”, eliminating reliance on external visual systems for localization and enabling full autonomy through tight integration of onboard miniaturized electronics, power systems, and computationally efficient perception, SLAM, and path‐planning algorithms. Additionally, we intend to enhance the robot's adaptability by optimizing adhesion mechanisms through redundant strategies suited for multiple terrain types, and by improving actuator designs to enable autonomous ground‐to‐wall transitions using a single module. Ultimately, these improvements aim to realize a fully untethered SPARC capable of autonomous 3D locomotion, encompassing trajectory tracking on both horizontal and vertical surfaces, as well as seamless transitions between different surface orientations.

## 4 Experimental Section

### Fabrication of the Origami Chambers

*CAD Modeling*

The computer‐aided design (CAD) model for the Kresling origami actuator was developed using commercial software (Solidworks 2021, Dassault Systems Inc.). The process begins with the creation of a triangular, faceted piece without thickness (Figure S8a, Supporting Information). Drawing inspiration from the Kresling origami pattern, these facets were then assembled onto a hexagonal base, meticulously piecing together each facet. Upon completion of this assembly, the facets were interconnected and subsequently thickened to impart structural integrity. To minimize deformation across the facets and localize it at the creases, additional minuscule sheets are strategically affixed to the external surfaces of the thickened facets (Figure S8b, Supporting Information). This innovative microstructural design ensured precise folding along the creases, enhancing the actuator's functionality. The specific parameters utilized in the CAD model are detailed in Table S1 (Supporting Information).

*3D Printing of Kresling Origami*

A 3D printing technique was introduced to fabricate the Kresling origami. This technique utilized a commercial fused deposition modeling (FDM) 3D printer (E2, Raise3D Inc.) (Figure S5, Supporting Information). This method employed thermoplastic elastomer (TPE, eSun, 83A) as the soft material for the actuator's soft body and polylactic acid (PLA, Raise 3D) as the rigid material for the rigid rings. A challenge encountered in FDM printing was airtightness. To mitigate this issue and enhance the overall success rate of the printing process, specific strategies had been adopted, including the adjustment of retraction settings and the printing speed, had been adopted, detailed in Table S2 (Supporting Information). During the 3D printing slicing process, the chamber was positioned vertically and adjust the support structure angle to a higher degree to prevent the generation of supports. This method effectively avoids damage to the surface of the origami chambers.

### Numerical Simulation

To accurately model the intricate geometry and thin‐walled structure of the Kresling‐pattern origami, HyperMesh (Altair) for mesh generation was employed. Initially, the origami CAD model was imported into HyperMesh, where 2D surface meshing was performed to create a triangular planar mesh, selecting a cell size of 0.4 mm. Ensuring mesh quality was crucial to avoid defects like collapse or deformation that could compromise analysis results. Therefore, during the quality assessment, meshes exhibiting aspect ratios greater than three or distortions above 60% were excluded to maintain integrity. Following the establishment of a 2D mesh meeting the quality standards and forming a closed envelope, a 3D mesh was generated. This step was essential to accurately mimic the solid structure of the origami chamber. For the nonlinear finite element analysis, the ten‐node modified quadratic tetrahedral cell (C3D10MH), which supports hybridization equations and linear pressure settings, was selected to construct the 3D mesh. The meshed model was then imported into ABAQUS (Dassault Systems Inc.) for simulation. Material properties were defined using the two‐parameter Mooney‐Rivlin hyperelastic model, derived from uniaxial tensile tests (see “Uniaxial tensile tests” in Supporting Information). To remove the rigid body translation and rotation of the pneumatic chamber, a full constraint boundary condition (*u*_*x*_ = *u*_*y*_ = *u*_*z*_ = *u*_*Rx*_ = *u*_*Ry*_ = *u*_*Rz*_ = 0) was applied to its open side. The internal vacuum was simulated by applying a static pressure of ‐80 kPa to the chamber's inner surface. Furthermore, to accurately represent internal rigid plates, rigid body constraints were applied within each hexagonal segment of the origami chamber, aligned with the physical dimensions of the plates. This comprehensive setup facilitates the finite element simulation of the chamber's motion under negative pressure, providing valuable insights into its behavior and performance (Movie S1, Supporting Information).

### Gait Control Algorithm

By leveraging the crawling motion gait and the pure pursuit controller, a gait control algorithm (Figure 3b) was developed. Algorithm S1 (Supporting Information) demonstrates the gait controller at the i‐th step, namely $F_{1}$. Tables S4 and S5 (Supporting Information) present explanatory descriptions for the symbols appearing in Algorithm 1 and Algorithm S1 (Supporting Information), respectively. To provide a more comprehensive description of the gait controller, it had been divided into the following four components:

1) Pure pursuit controller (line 1 ‐ 6): A target waypoint was selected on the reference path and fit an arc from the forefoot to that point to get the next step position in this pursuit controller. First, a circle was drawn using the forefoot as the center with a radius of *d* (Figure 3b). Parameter *d* (= 31.4 mm) was optimized to strike a balance between reducing the number of execution steps and enhancing trajectory tracking accuracy (see “Forward‐looking distance *d* optimization” in Supporting Information); if set excessively large, it could lead to considerable deviation from the trajectory, while a value that is too small may hinder the ability to navigate sharp turns. In the set of points ($P_{\text{goal}}^{i}$) along the reference trajectory within the circle, the target point $P_{\text{goal}}^{i}$ that is closest to the endpoint $P_{\text{final}}$ of the trajectory (Step I) was found. Next, an arc curve $L_{pp}^{i}$ was fitted between the target point and the forefoot position $P_{1}^{i}$, such that the tangent direction of the arc at the forefoot position aligns with the robot's orientation $z^{\prime i}$ (Step II). The aforementioned geometric relationship can be summarized by the following formula:
(4) $$\begin{array}{c} \left\{\begin{array}{c} \left\|\vec{P_{o}^{i}P_{1}^{i}}\right\|=\left\|\vec{P_{o}^{i}P_{\text{goal}}^{i}}\right\|=R\\ \vec{P_{o}^{i}P_{1}^{i}}\cdot z^{\prime i}=0\\ ∠P_{1}^{i}P_{o}^{i}P_{\text{goal}}^{i}=\Omega^{i} \end{array}\right. \end{array}$$
  where $P_{o}^{i}$ is the center of $L_{pp}^{i}$.
  For formula (4.3), it can be transformed into the relationship between the forward distance *d*, the central angle Ω^*i*^, and the radius *R* of the arc, which is as follows:
(5) $$\frac{d}{2R^{i}}=\sin\left(\frac{\Omega^{i}}{2}\right)$$
  In Equation (4), the unknowns are the position of the center $P_{o}^{i}\left(x,y\right)$, the radius *R* ^*i*^, and the central angle Ω^*i*^. This system of equations was statically determinate and has a unique solution. The solution for $L_{pp}^{i}$ was summarized in Algorithm S2 (Supporting Information), namely $F_{2}$. The corresponding symbol explanation can be found in Table S6 (Supporting Information).
2) Inverse kinematic model (line 7 ‐ 13): The workspace of the robot's forefoot was used to help determine the landing point of the forefoot in the next time step. This was achieved by applying the piecewise constant curvature assumption and the inverse kinematic model. As illustrated in Figure 3b, the intersection point of the fitted arc with the contour of the forefoot's workspace was identified as the landing point ${\hat{P}}_{1}^{i+1}$ for the forefoot, marked as a star. Using the inverse kinematic model (the Equation (2)), the lengths of the three actuators $l^{i+1}$ at this landing point were determined. The solution for $l^{i+1}$ is summarized in Algorithm S3 (Supporting Information), denoted as $F_{3}$. It was important to note that under a pure tracking controller, there was no guarantee that the landing point of the robot's forefoot in each step would be precisely placed on the reference trajectory.
3) Self‐sensing model (line 14 ‐ 23): The lengths of the three actuators were converted into their corresponding self‐twisting angles $\phi^{i+1}$ using the Kresling‐origami self‐sensing model and Newton's method. By applying vacuum to the three Kresling origami actuators accordingly and using a simple PID control loop with the calculated twisting angles as the feedback signal, the forefoot moves to the target position.
4) Shuffling and sensing (line 24 ‐ 27): With the forefoot secured and all three chambers contracted to their shortest lengths, followed by securing the hindfoot, the motion for step *i* + 1 was completed. After completing the motion, the OptiTrack motion capture system was used to capture the position and orientation of the robot's forefoot. This data served as the initial state for the next cycle.

In summary, Algorithm 1 provided a high‐level closed‐loop control mechanism by utilizing a tracking camera to continuously update the real‐time position of SPARC, enabling it to navigate along the reference trajectory until reaching the endpoint. The PID controller in Algorithm S3 (Supporting Information) serves as the low‐level closed‐loop control. It utilized encoder feedback on the angle to regulate the actuator length, thus controlling the motion and state of SPARC. Thanks to this robust and sophisticated dual‐closed‐loop control system, the robot could autonomously navigate along a predefined trajectory. Figure S6 (Supporting Information) illustrates the connection and control signal flow within SPARC's electronic system. In this schematic, the red, green, and yellow lines depict the signal flows for the three origami actuators, respectively, while the purple and gray lines represent the fore and hind feet. Additionally, solid lines indicate electrical signal flows, and dashed lines signify air flows.

### Experimental Settings and Setups

In the experiment, a motion capture system (OptiTrack Prime41) was utilized to measure the global position of SPARC. The master controller (Jetson Nano, Nvidia) received the position data and the predefined trajectory and calculates the desired twisting angles for the three origami actuators using built‐in algorithms. This data was transmitted to the slave controller (Arduino Mega 2560), which controlled the actuation of the three origami actuators. Three proportional electrovacuum regulators (ITV209, SMC) were used to regulate the input negative pressure, controlled synchronously with pulse width modulation signals from the slave controller. A digital‐to‐analog converter module (DC2025A‐A, ADI) ensured stable voltage input between the slave controller and the regulators. The signals generated by the slave controller for driving the actuators were transmitted via the I2C bus protocol, ensuring synchronized movements of the three actuators. As feedback, three angle encoders (AS5600) measure the torsion angles at the ends of the actuators and transmit this information back to the slave controller. For controlling the adhesive foot, the slave controller sent a digital signal to the vacuum suction cups through relays (Risym) and a solenoid valve (AirTAC 3V2). The physical layout of the described system is detailed in Figure S6 (Supporting Information).

## Conflict of Interest

The authors declare no conflict of interest.

## Author Contributions

W.F. and J.W. designed the study, supervised data collection, and contributed the whole manuscript preparation and design. J.W., H.Z., Q.W., and Q.H. carried out the experiments. W.F., J.W., and X.H. wrote the manuscript. G.C., H.W., and X.H. directed the research.

## Supporting information

Supporting Information

Supplemental Movie 1

Supplemental Movie 2

Supplemental Movie 3

Supplemental Movie 4

Supplemental Movie 5

Supplemental Movie 6

## Data Availability

The authors declare that all data supporting the findings of this study are available with the article, its Supplementary Information files and the following repositories: https://github.com/UMich‐HDRLab/SPARC.
