# Supplementary material for: SPARC: A Soft, Proprioceptive, Agile Robot for 3D Climbing and Exploration with Precise Trajectory Following
Source: Adv Sci (Weinh). 2025 Sep 14;12(41):e10382. doi: 10.1002/advs.202510382 (PMC12591128; doi:10.1002/advs.202510382)
Supplement: Supplementary file 1 — Supporting Information [file ADVS-12-e10382-s006.pdf]

Supplementary Materials

Geometrical analysis of the Kresling origami

We define the basic Kresling origami element by the variables a, b and α, which represent the length of the hexagon side, the length of the mountain-shaped crease AA', and the angle between the mountain and valley creases, respectively (**Figure S8a,c**, and **Table S1**). Therefore, the radius r of the circumcircle of the hexagon can be expressed as follows:

$$r = \frac{a}{2\sin\left(\frac{\pi}{6}\right)} \tag{S1}$$

The folding state of the single-section origami chamber is described using the axial rotation angle ϕ and the height h. Point A'' is the projection of A' on the OAB plane, thus $\angle AOA'' = \phi$ and $OO' = h$. Due to the geometric constraints of the inner hard material hexagon, $O'A' = r$ keeps constant during the folding process. Assuming that the origami structure keeps axial folding, then $OA'' = O'A' = r$, $A'A'' = OO' = h$. The lengths of AA'' and h can be derived from $\triangle OA''A$ and $\triangle AA''A'$ (**Figure S8c**).

$$AA'' = c = 2r\sin\frac{\phi}{2} \tag{S2}$$

$$h = \left(b^2 + 2r^2(\cos\phi - 1)\right)^{1/2} \tag{S3}$$

Eq. S3 reveals the kinematic relation between the axial length h and the axial twisting angle ϕ of the origami structure. This relation is governed by two geometric parameters: r and b. The parameter r is a constant, as given by **Eq. S1**. On the other hand, the parameter b is not a constant in our modified analytical model.

Conventional analytical models for the Kresling origami pattern used to assume that the mountain crease b, namely the length of AA' (**Figure S8b**), remains constant while contracting [73, 74]. Yet, with the 3D-printed origami chamber using soft material, the variation of b becomes significant. Therefore, the theoretical model requires modification.

In order to explore the variation principle of b with ϕ, we use the simulation value of h to calculate the true value of b, based on **Eq. S3**. From the data presented in **Figure 2b**, we fit b as a function of ϕ as

$$b = A_0 e^{-B_0\phi} + C_0 \tag{S4}$$

Combining **Eq. S3 and S4**, we obtain the modified analytical model of self-sensing chambers as

$$\begin{aligned} l = f(\Phi) = nh &= n\left(b^2 + 2r^2\left(\cos\frac{\Phi}{n} - 1\right)\right)^{1/2} \\ &= n\left(\left(A_0 e^{-B_0\frac{\Phi}{n}} + C_0\right)^2 + 2r^2\left(\cos\frac{\Phi}{n} - 1\right)\right)^{1/2} \end{aligned} \tag{S5}$$

where l is the length of the actuator, Φ is the axial twisting angle, $n(=6)$ is the number of Kresling sections, h is the height of each section of Kresling origami, b and r represent the length of the mountain-shaped crease and the radius of the circumcircle of the hexagon, and A_0, B_0, C_0 are the fitting coefficients. We list the constants of the above parameters in **Table S7**.

Uniaxial tensile test

We conducted uniaxial tensile tests on TPE samples after 3D printing to account for possible alterations in material properties due to the 3D printing process. **Figure S9a** displays the specimens and the experimental procedure for uniaxial tensile testing. We used three crosswise and three longitudinal specimens for the experiments. As illustrated in **Figure S9b**, the two different printing patterns had minimal impact on the stress-stretch curve of the material. We tested the tensile stretch of TPE up to 6, which is beyond the operational range of the robot, yet the material did not reach its yield or rupture point, demonstrating TPE's high stretchability. To describe the hyper-elastic deformation behavior of TPE accurately, we applied a two-parameter Mooney–Rivlin hyper-elastic material model [75, 76] that fitted the average stress–stretch data well. The model is characterized by the following relationship:

$$\sigma = 2\left(\lambda^2 - \lambda^{-1}\right)\left(C_{01} + C_{10}\lambda^{-1}\right) \tag{S6}$$

In this equation, σ denotes stress, while λ represents the stretch ratio. The parameters used in this model are listed in **Table.** S3. This model was calibrated to match the experimental stress-strain data utilizing the curve fitting tools available in ABAQUS.

Wall thickness optimization of the Kresling origami actuator by simulation

Based on experimental observations, we identified the hexagonal region of the Kresling origami actuator as the most failure-prone area (the leakage region) under contraction. Assuming this region experiences the maximum stress, we used a design yield criterion based on material properties to guide our simulations. Specifically, for thermoplastic elastomer like TPE 83A we used here, engineers and scientists usually treat **100% modulus** as the "design yield" for elastomeric parts based on ASTM D412 standards. It marks a reproducible point on the stress–strain curve that falls neatly within the elastic–viscoelastic region, but before significant strain hardening or damage occurs. Therefore, we define the specific value as the 100% modulus of TPE 83A (**4.01 MPa**, Figure S9b) and carry out a parametric finite-element sweep in Abaqus, with thickness varied from 0.4 mm to 0.9 mm (in 0.1 mm increments) to determine the thickness at which maximum stress under -60 kPa vaccum equals the 100% modulus. The workflow is as follows: (i) generate each CAD model in SolidWorks (Section 4.1.1), and (ii) import the model into Abaqus to perform non-linear analysis (Section 4.2). The finite element analysis is shown in Figure S14a and Figure S14b summarises the resulting maximum stress as a function of thickness under -60 kPa. The corresponding data are shown in Table S8. As shown in Figure S14b, as the wall thickness increases from 0.4 mm to 0.9 mm, the maximum stress under -60 kPa decreases. When the wall thickness reaches 0.68 mm, the maximum stress equals 4.01 MPa, which corresponds to the 100% modulus of TPE 83A. Therefore, the optimal thickness, as determined by simulation, is 0.68 mm. Experimentally, we use a wall thickness of 0.6 mm. Although it is slightly below the simulated optimal thickness, the actuators have been shown to endure over 20,000 actuation cycles without failure (Figure S3), thanks to our meticulously tuned fabrication parameters, delivering near-optimal performance.

Optimization of Kresling origami parameters using finite element analysis

A key performance metric for our robotic system is the SPRAC's workspace and bending angle (Figure 2f–g), which, as shown through kinematic analysis (Section 2.4), directly scale with the actuator's contraction ratio. Therefore, we focused on maximizing this contraction by optimizing the geometric parameters of the Kresling cell-specifically, a, b, and α. Due to geometric constraints, the angle α is fixed at 30 degrees for a regular hexagonal base, and edge length a serves as a design parameter correlated with the overall device dimensions; therefore, we fix a as 15 mm for our robot. The optimization thus reduces to finding the b value that maximizes contraction. For convenience, we introduce the triangle-facet height h_0 (Figure S15b); once the optimal h_0 is known, the corresponding b follows directly from the geometric relations. We performed a parametric finite-element sweep in Abaqus, varying h_0 from 15 mm to 25 mm in 1 mm increments. The workflow includes (i) generating CAD models in SolidWorks (Section 4.1.1), and (ii) importing the model into Abaqus and running non-linear simulations (Section 4.2). The FEA results are shown in Figure S15a and Figure S16a. We demonstrate the original states and actuated states at -60 kPa for the full range of h_0. The relationships between contraction displacement and vacuum are plotted in Figure S15d and Figure S16b, while Figure S16c summarises the resulting contraction ratio as a function of h_0. The corresponding data are shown in Table S9. As shown in Figure S16c, when h_0 increases from 15 mm to 21 mm, the contraction ratio gradually increases. This is because at smaller h_0, the Kresling origami actuator's original length is short, and the valley crease angle–defined as the angle between $\triangle A'AB$ and $\triangle A'B'B$ (See Figure S15c)–is correspondingly small. Under low vacuum, this angle quickly approaches zero, causing adjacent facets to come into contact, which limits further contraction even if additional vacuum is applied. For example, as shown in Figure S15d, when $h_0 = 15mm$, the contraction displacement plateaus between –50 kPa and –60 kPa. However, as h_0 increases from 21 mm to 25 mm, the contraction ratio sharply decreases. In this range, the valley crease angle remains above zero, and adjacent facets do not contact each other under -60 kPa. Additionally, as h_0 increases, the triangle facet of the Kresling unit becomes more perpendicular to the base, causing the pressure-induced force to be directed more parallel to the base. This results in greater facet deformation rather than axial contraction. We also observed that at larger h_0 values, the Kresling origami actuator becomes more prone to instability; that is, individual Kresling units within the same actuator exhibit varying contraction ratios (see Figure S16(a, $h_0 = 23mm$ and $24mm$)), contrary to the assumption of uniform unit behavior. This variability increases the control complexity of the robotic system. Similar findings are reported in [77]. Based on these findings, the optimal simulated value was $h_0 = 21mm(b = 21.63mm)$ (Table S9). In experiments, we selected h_0 = 20 mm (b = 20.81 mm), which delivered 5% below maximum contraction while ensuring reliable fabrication and operation.

Reliable adhesion based on preloaded-force strategy

To achieve reliable and consistent adhesion on smooth surfaces, we employed a strategy that applies a pre-loaded downward force to the suction cups, increasing the contact area at the suction cup margins and ensuring a secure seal. Specifically, as shown in Figure S12a, during the contraction process, the front feet are fixed while the three actuators begin to contract. Based on our kinematics modeling, we slightly overdrive the top actuator compared to the bottom two, creating a 5-degree downward bending angle. This preloads the hind suction cups, compressing and deforming them to maximize contact with the surface, thereby ensuring a reliable seal once vacuum is applied, anchoring the hind feet. Similarly, during the elongation process (Figure S12b), the hind feet are fixed, and the three actuators begin to extend. Again, by slightly overdriving the top actuator, we introduce a 5-degree downward tilt, preloading and deforming the front suction cups to ensure a secure seal. Through this controlled preload strategy, the suction cups reliably anchor to the surface upon vacuum activation.

Terrain adaptability and load carrying experiment

To validate SPARC's climbing performance under varying terrains and loads, we conducted experiments on four surfaces—dry glass, wet glass, wood, and acrylic—using five different load conditions (0 - 500g). In each trial, SPARC ascended three steps on a wall using identical open-loop control parameters. The recorded climbing speeds were averaged, and the final results showed a maximum deviation of only 8.9%.

As illustrated in Figure S11, SPARC consistently achieved stable climbing across all four terrains, demonstrating its robust adaptability to different surfaces. Among the tested surfaces, SPARC exhibits the highest climbing speed on acrylic and the lowest on wet glass. This discrepancy may be attributed to minor slippage of SPARC's suction cups on the tested surfaces. On wet glass, the cups tend to slide, reducing climbing speed. In contrast, the acrylic plates offer the greatest resistance to sliding, thereby minimizing speed loss and yielding the fastest climbing performance. Additionally, although the climbing speed decreased slightly as the load increased, SPARC maintained between 79% and 86% of its peak speed under maximum load. This indicates that added load has a minor impact on the robot's overall motion performance.

Pure pursuit controller

Comparison with rough gait controller

To assess the impact of the arc trajectory generated by the pure pursuit controller, we developed a rough gait controller and performed gait simulation comparisons using MATLAB. This controller comprises two main steps. Initially, it involves pinpointing the farthest point from the initial position along the trajectory within the forefoot's working range and then adjusting the forefoot to reach this position. Subsequently, the forefoot is immobilized, the actuator contracts and the hindfoot aligns with the trajectory.

Figure S10 a-f illustrate the simulation of SPARC's movement along square, circular, and sinusoidal paths using the rough controller. Specifically, **Figure S10 a-c** display the positions of the fore and hind feet, along with the robot's body, when all three chambers are at their minimum length after each movement step. Moreover, **Figure S10 d-f** depict the turning angles of the robot for each movement step. Although incorporating a rough controller, the robot accurately tracks the predefined trajectory; however, square and sinusoidal trajectories demand substantial turning angles at sharp turns. The maximum turning angle required for square trajectories is approximately 100 degrees, whereas for sinusoidal trajectories, it is around 80 degrees. During practical motion, the robot's inability to achieve turning angles exceeding 50 degrees leads to noticeable discrepancies between the actual landing positions of the forefoot and the theoretically calculated positions. Consequently, substantial deviations arise between the actual forefoot trajectory and the reference trajectory. When dealing with circular reference trajectories, the rough controller can effectively track the path with minimal turning angles. Consequently, the rough controller is deemed inadequate for trajectories with sharp curvature turns.

With a forward-looking distance of $d = 40$ mm, we conducted simulations of the pure pursuit controller for circular, square, and sinusoidal path tracking (**Figure S10 g-l**). The simulation outcomes demonstrate precise alignment between the robot's trajectory and the reference path governed by the pure pursuit controller. Moreover, this controller exhibits decreased turning angles per step compared to the rough controller. For square and sinusoidal trajectories, SPARC's maximum turning angle does not exceed 35 degrees. Moreover, implementing the pure pursuit controller only requires confirming an intersection between the forefoot and the reference trajectory within the forward-looking distance range at every step. When dealing with reference paths featuring sharp curvature turns, extending the forward-looking distance d enables the robot to preemptively adjust its posture and conform to the high-curvature reference trajectory. Consequently, the pure pursuit controller demonstrates enhanced robustness compared to the rough controller.

Forward-looking distance d optimization

In the aforementioned simulation calculations, we manually gave the forward-looking distance d as 40 mm. In fact, the forward-looking distance affects both the following accuracy and the total number of steps of the robot moving along the trajectory. In order to obtain a more appropriate forward-looking distance, we analyze the robot's motion gait by simulating it through Matlab. Since square trajectories contain right-angle turns, which is the main application scenario for pure tracking algorithms, we optimize SPARC's motion gait in square trajectories. We set the objective function as:

$$g(d) = \frac{E_{\text{sum}}(d)}{\max_{i=d_{\text{min}}}^{d_{\text{max}}} \{E_{\text{sum}}(i)\}} + \frac{N_{\text{step}}(d)}{\max_{i=d_{\text{min}}}^{d_{\text{max}}} \{N_{\text{step}}(i)\}} \tag{S7}$$

where E_{sum} represents the sum of the distance errors between the forefoot landing point and the trajectory deviation for each step to walk the whole trajectory under the SPARC's trajectory tracking algorithm, and N_{step} represents the number of steps required for SPARC to travel the entire trajectory.

Through an exhaustive search over parameter d with 0.1-mm increments, we identified the global minimum of function $g(d)$, as well as the forward-looking distance d^* at this point. As shown in Figure S13, we repeated the above procedure for square trajectories with different side lengths to obtain a set of optimal forward-looking distances $\{d^*\}$. Finally, we adopted their average value $d^*_{\text{mean}} = 31.4$ mm as the optimization result of the forward-looking distance.

Trajectory design

1) **S-shape curve:** This trajectory consists of two semicircles in opposite directions, each with a diameter of 30 cm, resulting in a total length of 94.25 cm. It evaluates SPARC's ability to follow circular paths in different directions.
2) **S-shape square:** This trajectory includes two squares in opposite directions, each with a side length of 30 cm, totaling a length of 150 cm. It assesses SPARC's ability to navigate trajectories with sharp right-angle turns in different directions.
3) **Vertical semicircle:** This trajectory features a semicircle located on a vertical wall, with a diameter of 30 cm and a total length of 47.12 cm. It is used to assess SPARC's ability to follow non-linear trajectories on a vertical surface, a challenge for current soft climbing robots.
4) **Vertical line:** This trajectory involves a straight line on a vertical wall, with a total length of 21.5 cm. Notably, SPARC carried a payload of 500 g, more than twice its body weight (210 g), while following this trajectory.

Trajectory error calculation

We propose a method for assessing errors in mobile robots by utilizing data from the landing point of the forefoot and its subsequent trajectory. In every step, we identify the point on the trajectory that is nearest to the forefoot's landing point, determine the Euclidean distance between them, and regard this distance as the deviation for that particular step. We denote the average deviation and relative average deviation of the entire trajectory as follows:

$$\begin{cases} E_{\text{ave}} = \sqrt{\frac{\sum_{i=1}^{M} e_i^2}{M}} \\ \delta = \frac{E_{\text{ave}}}{L_{\text{traj}}} \end{cases} \tag{S8}$$

where E_{ave} denotes the trajectory's average deviation, e_i signifies the minimum distance from the fore foot's landing point for the i-th step to the trajectory, M indicates the total number of steps taken by the robot along this path, δ denotes the relative average deviation, and l_{traj} represents the overall length of the trajectory.

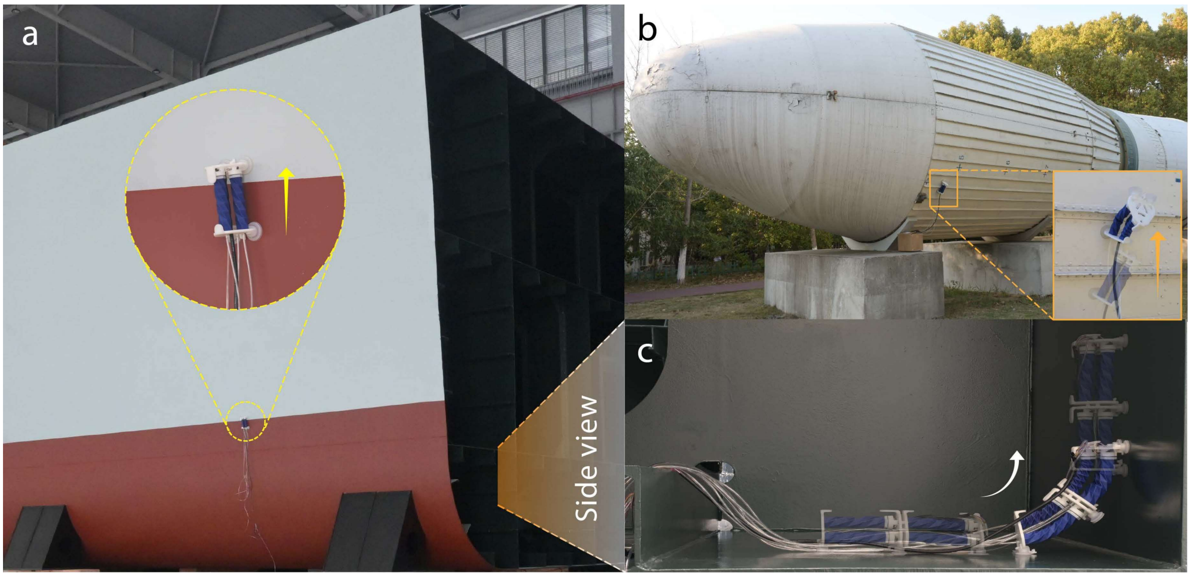

Figure S1. Examples of SPARC moving in real scenarios: (**a**) and (**c**) show SPARC climbing the exterior of a ship section and navigating transitions within the ship's interior, respectively, while (**b**) depicts SPARC ascending a rocket's outer surface, crossing structural ribs.

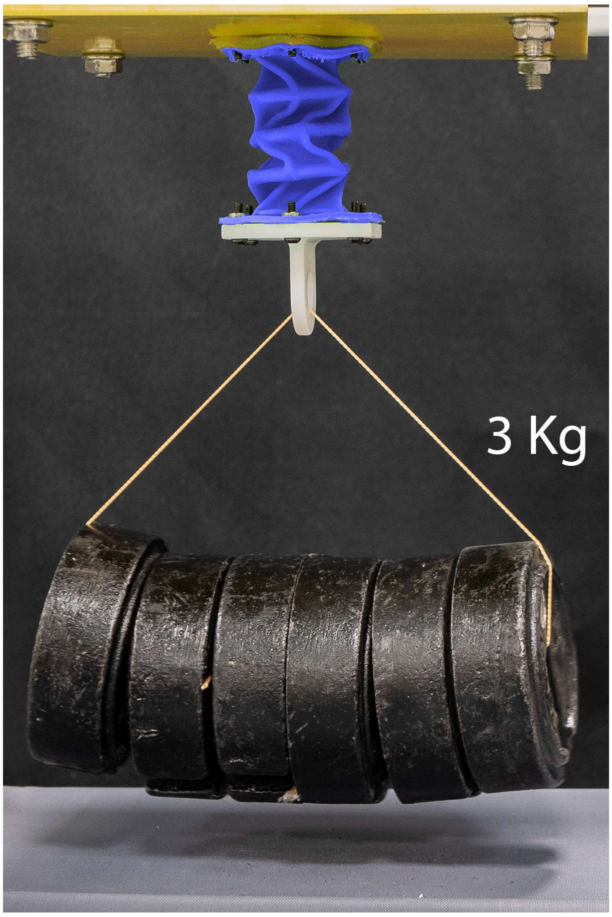

Figure S2. Payload test of proposed Kresling origami actuators. The origami actuator can lift a 3 Kg weight, with 15 g self-weight (more than 200 times).

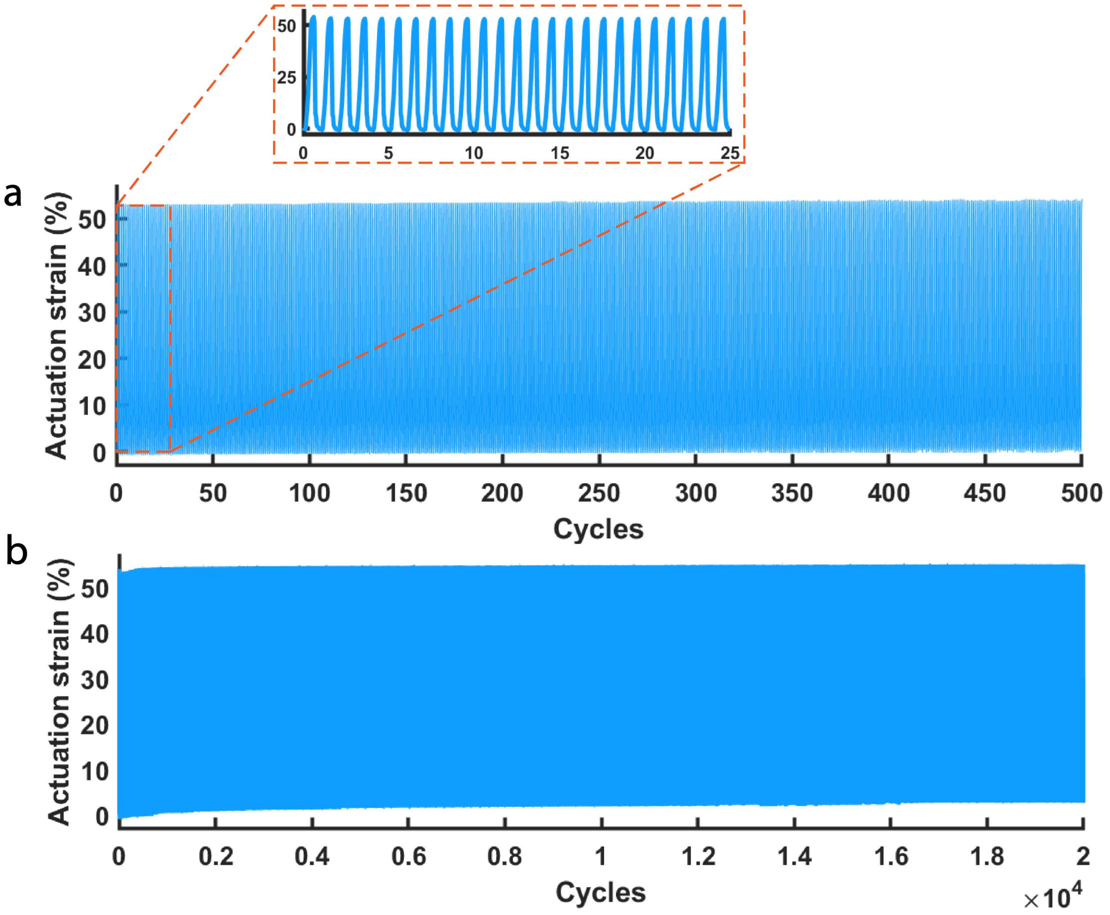

Figure S3. Cyclic test of the Kresling origami actuators. a, First 500 cycles, with a zoomed-in view of initial 25 cycles. **b**, 20000-cycle lifetime test showing first-cycle effects and actuation creep. All tests were conducted at a cycle frequency of 1 Hz.

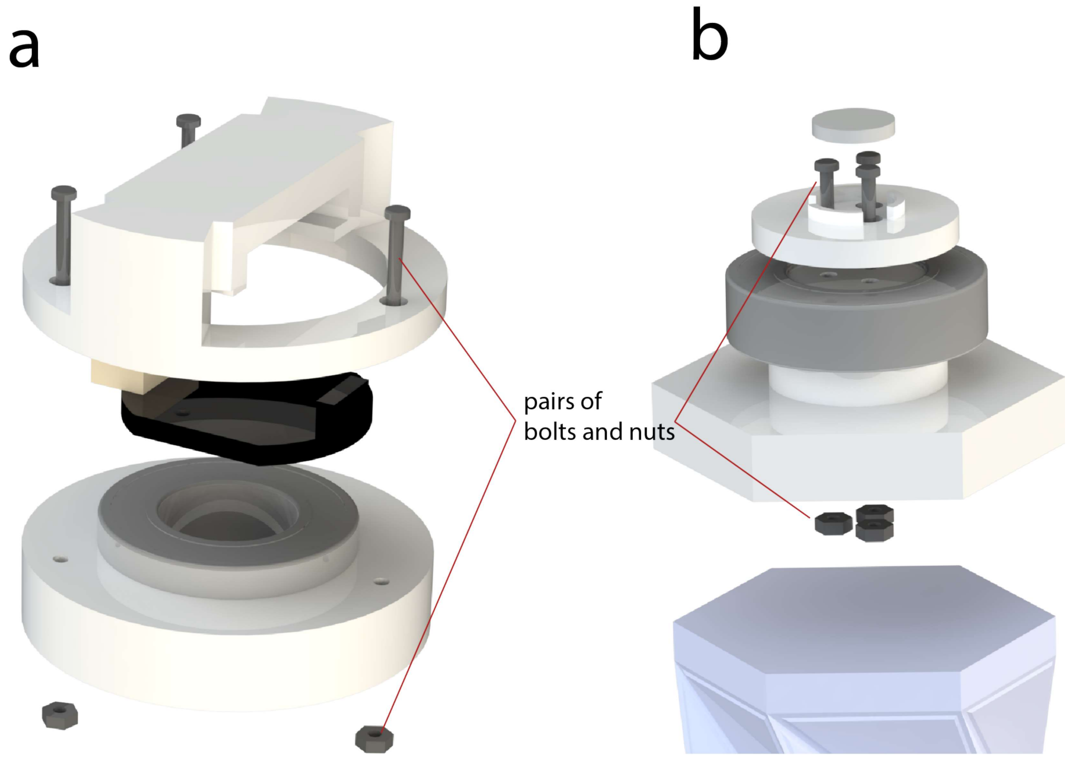

Figure S4. Exploded view of the bearing assembly at the distal end of the Kresling origami actuator. a, schematic of the bearing outer ring connection to the forefoot base. **b**, schematic of the bearing inner ring connection to the Kresling origami chamber end.

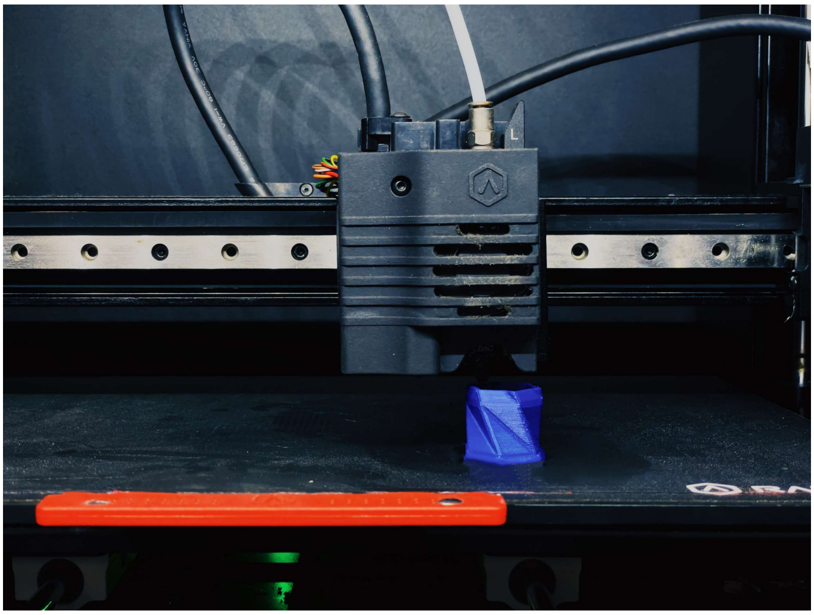

Figure S5. Fused deposition modeling (FDM) 3D printing of the Kresling origami actuators.

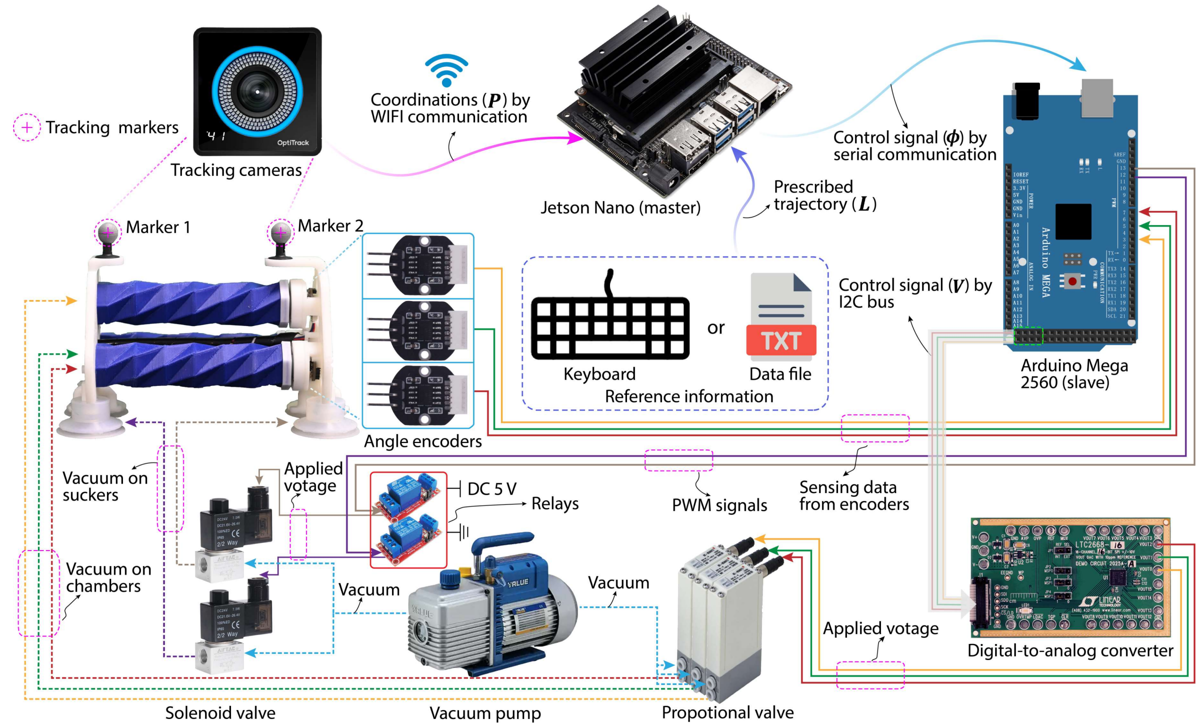

Figure S6. Experimental setup for the dual closed-loop control of SPARC. The flow lines represent the signal pathways for the three origami actuators, distinguished by red, green, and yellow lines, as well as the fore and hind feet, represented respectively by purple and gray lines. Solid lines indicate the flow of electric signals, while dashed lines depict the flow of air signals.

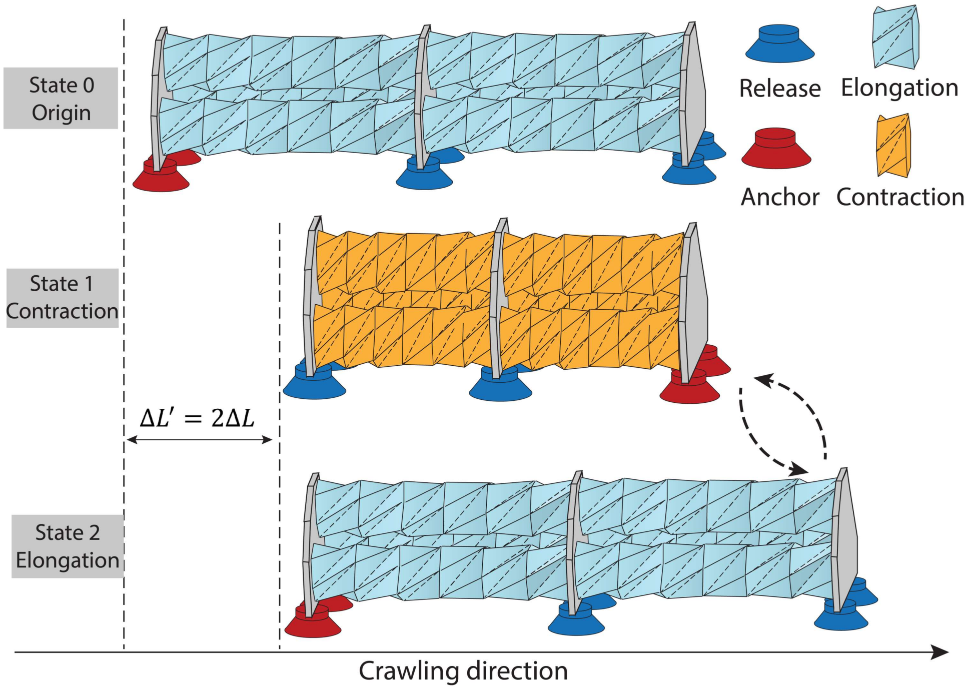

Figure S7. Crawling motion of the serial SPARC. Starting with the initial state, a forward gait cycle consists of a sequence of forefoot anchored, chambers contraction, hindfoot anchored, and chambers extension.

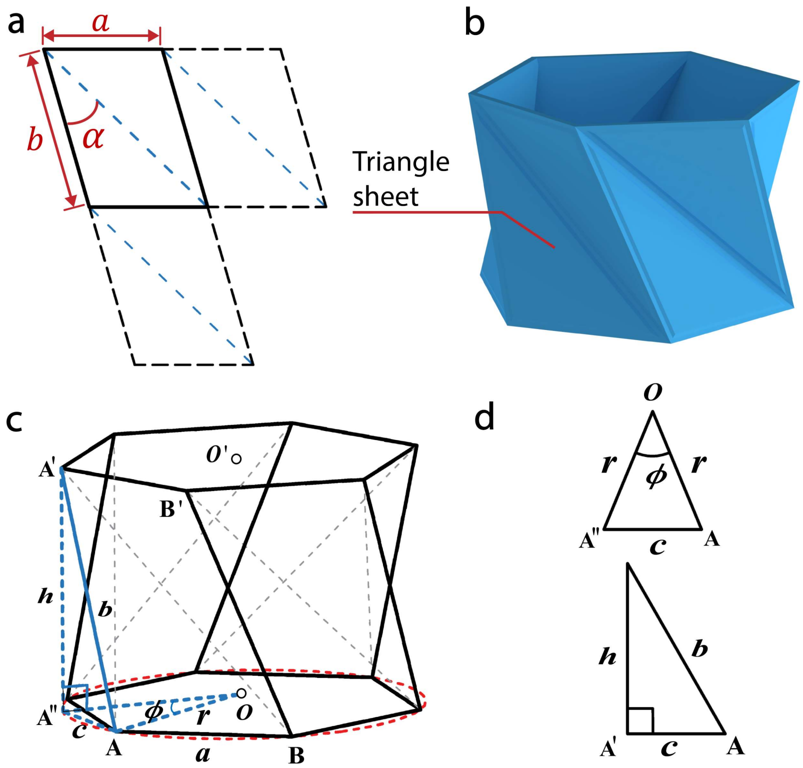

Figure S8. Design and geometric model of Kresling unit. a, Planar base crease pattern of Kresling origami and its dimensions. **b**, Triangle sheet on the facet. **c**, Geometric model of the Kresling origami unit. A'' is the projection of A' on the base plane. **d**, Front view of two triangles $\triangle OA''A$ and $Rt\triangle AA''A'$

.

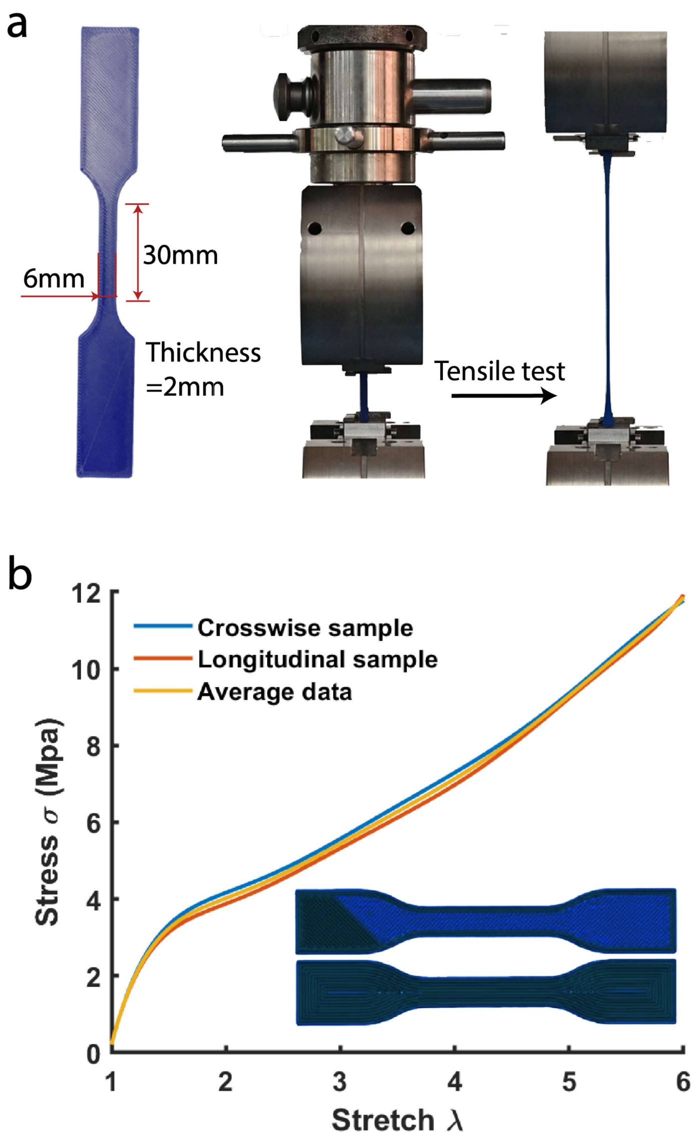

Figure S9. Uniaxial tensile testing experiments. b, Uniaxial tensile test results for specimens with different printing textures.

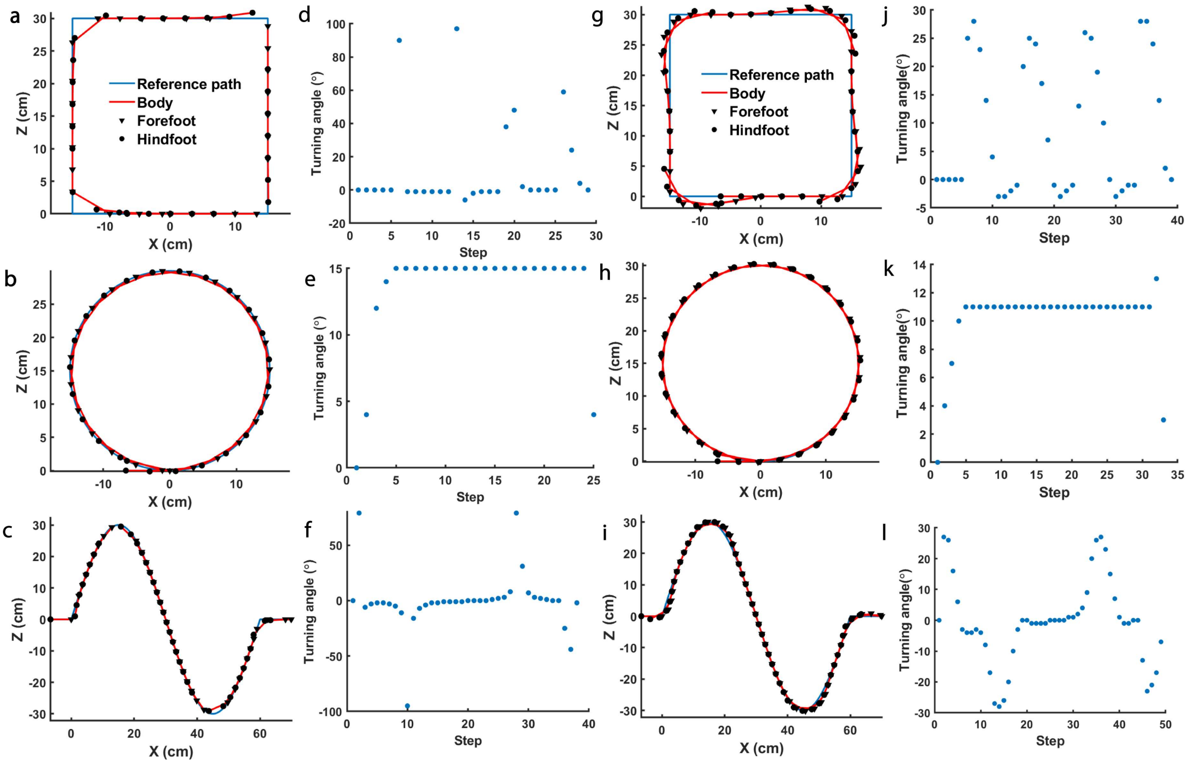

Figure S10. Comparison between a rough controller and the proposed pure pursuit controller through gait simulations using three types of curves: square, circular, and sinusoidal shapes. a-f, Path following and turning angle simulations for the rough controller. **g-l**, Path following and turning angle simulations for the pure pursuit controller.

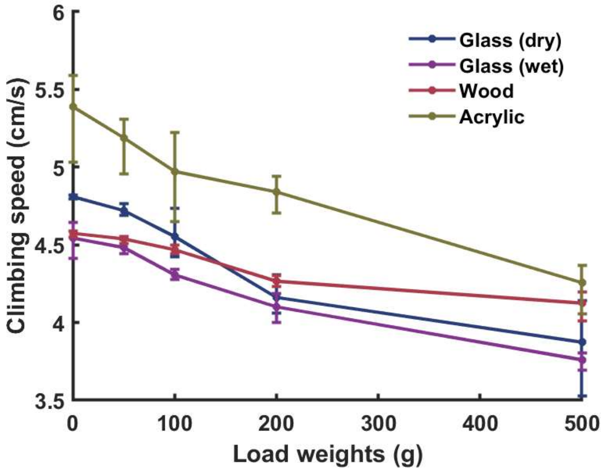

Figure S11. Comparison of climbing speeds across four wall surfaces—dry glass, wet glass, wood, and acrylic—under varying payload conditions.

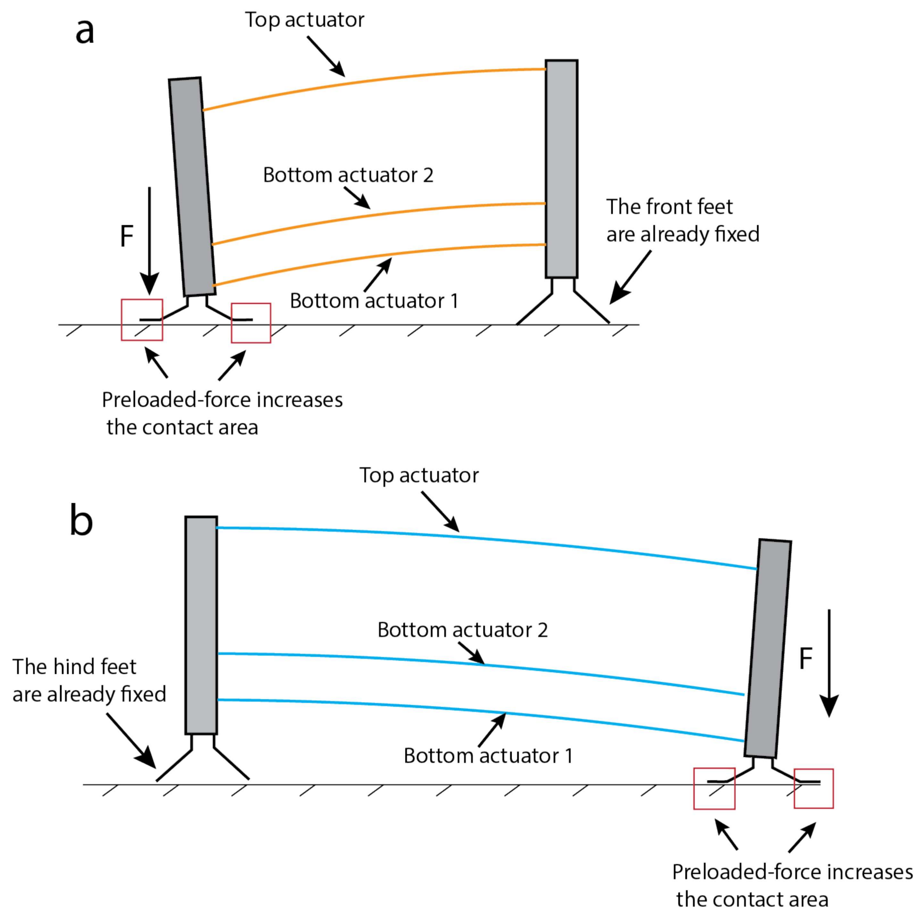

Figure S12. Reliable adhesion based on preloaded-force strategy. a, contraction process. b, elongation process.

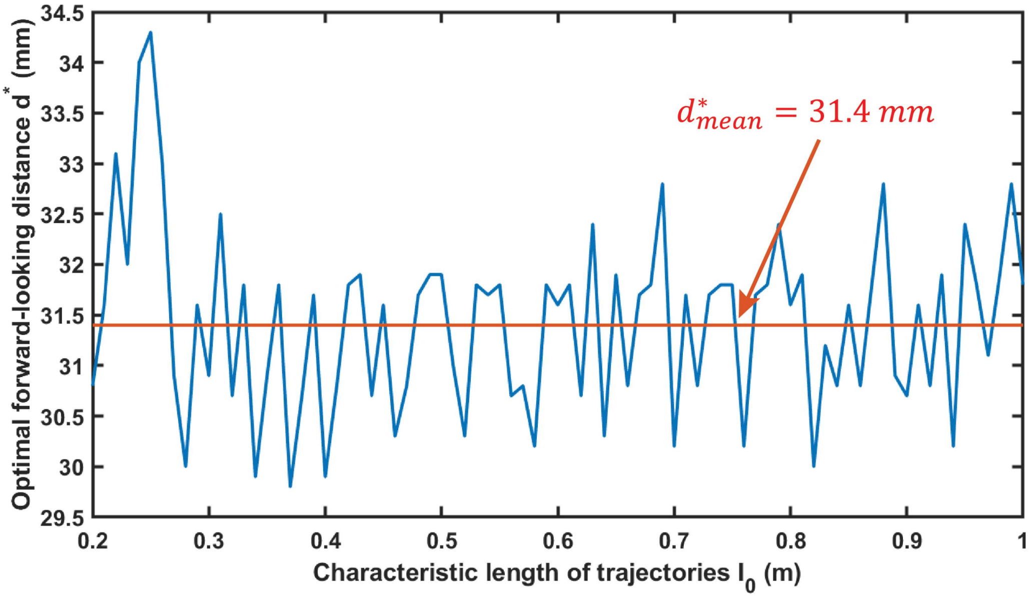

Figure S13. The optimal forward-looking distances for trajectories of varying sizes.

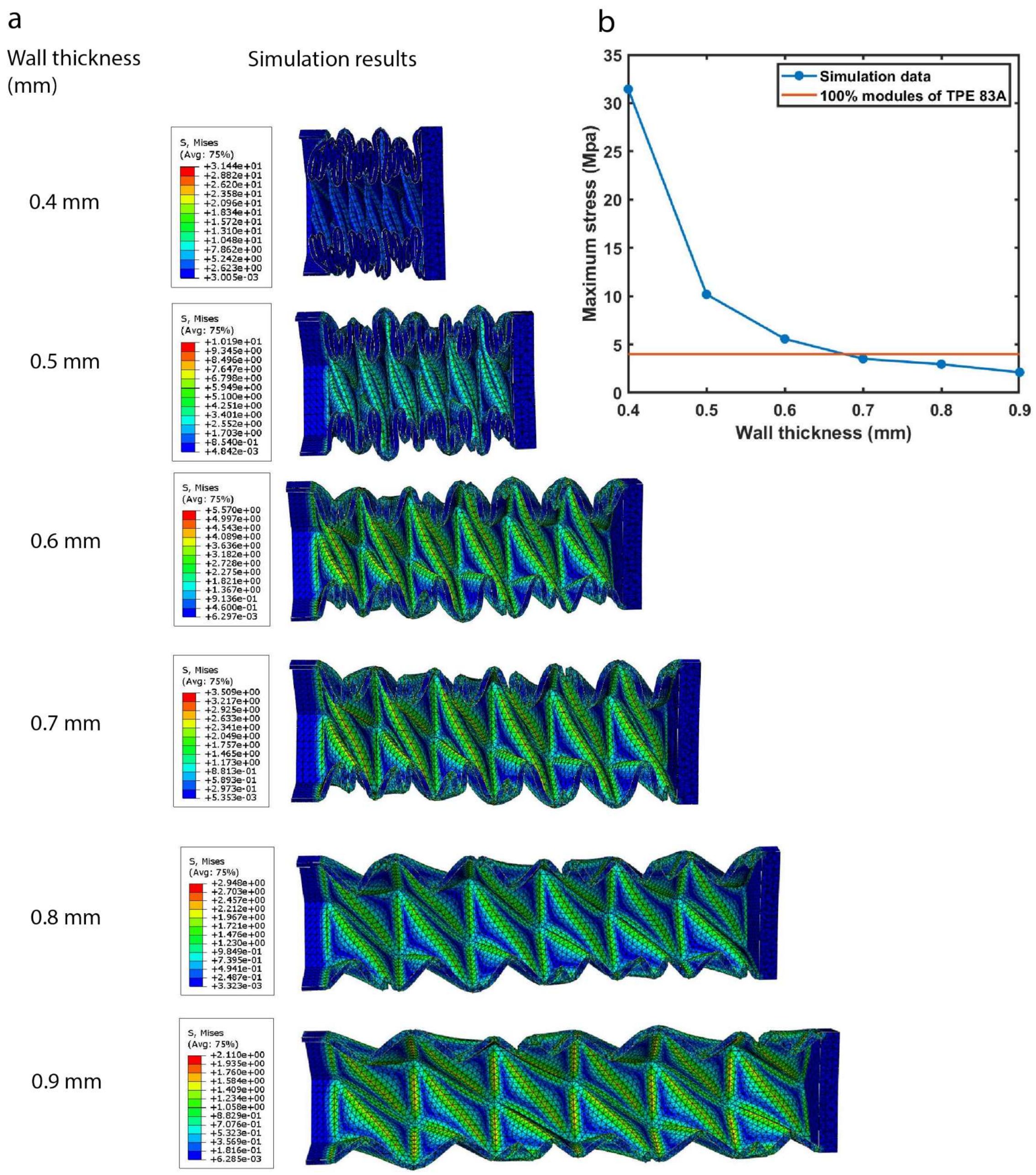

Figure S14. Finite element analysis (FEA) simulation for wall thickness optimization of the Kresling origami actuator. a, FEA results showing the internal wall stress profiles under –60 kPa for wall thicknesses ranging from 0.4 mm to 0.9 mm. **b**, maximum stress as a function of wall thickness under –60 kPa.

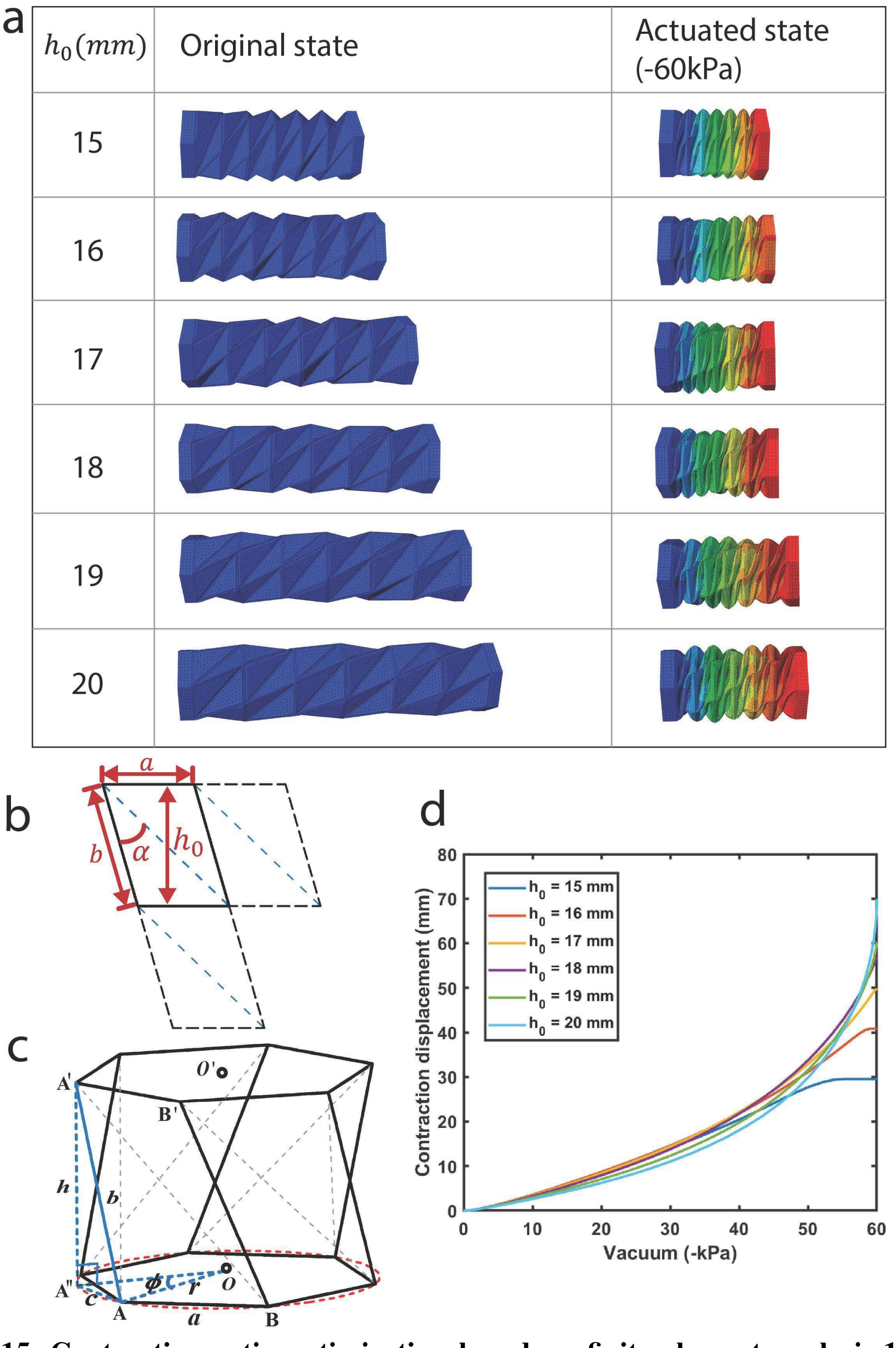

Figure S15. Contraction ratio optimization based on finite element analysis-1 a, FEA results with h_0 varied from 15 mm to 20 mm. **b**, Planar schematic of the basic unit of Kresling origami and its dimensions. **c**, Geometric structure of a single Kresling origami unit. **d**, Contraction displacement versus vacuum with h_0 varied from 15 mm to 20 mm.

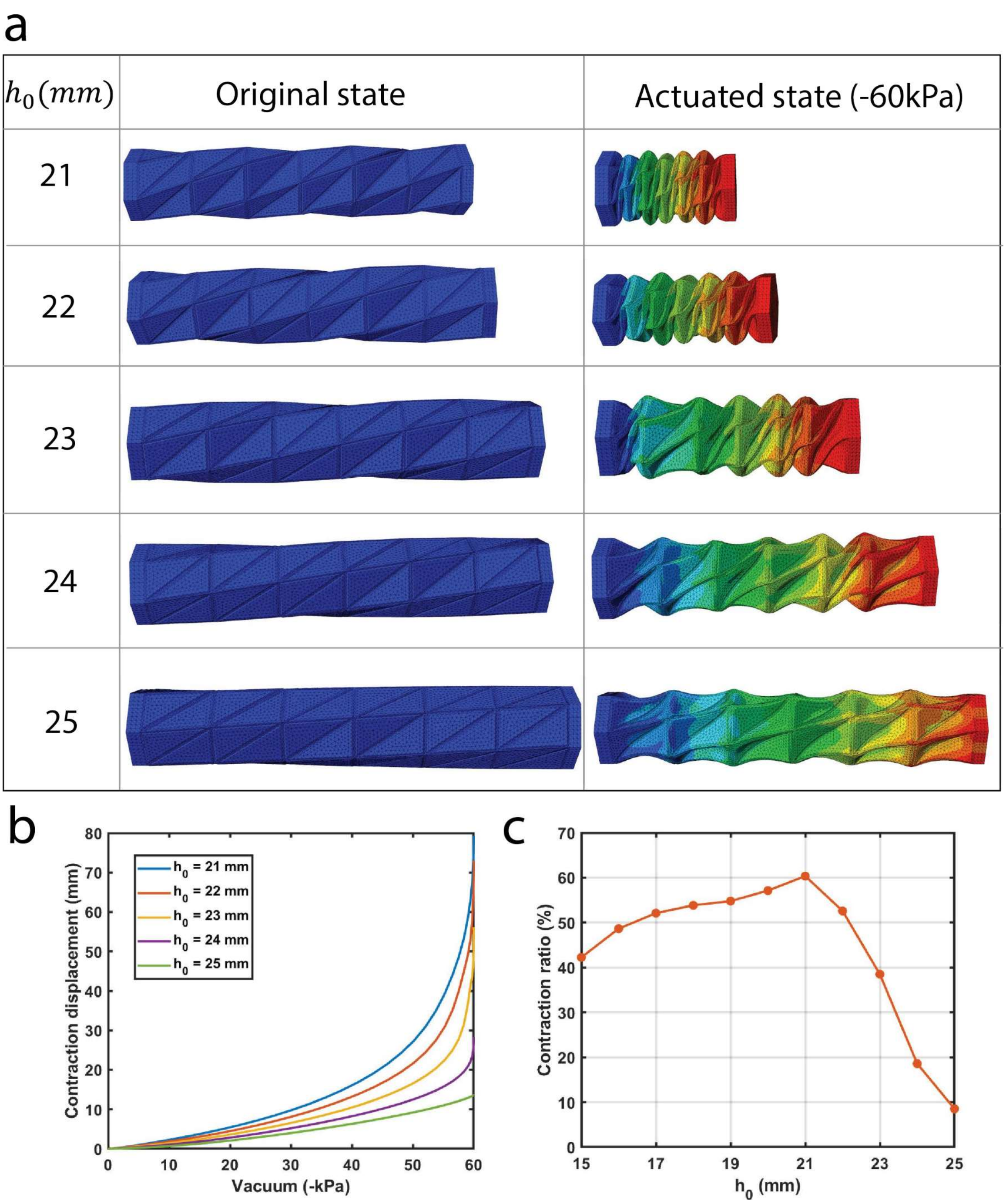

Figure S16. Contraction ratio optimization based on finite element analysis-2 a, FEA results with h_0 varied from 21 mm to 25 mm. **b**, Contraction displacement versus Vacuum with h_0 varied from 21 mm to 25 mm. **c**, Relationship between contraction ratio and h_0.

Table S1. Dimensions of the origami chamber

Symbol	Description	Value
a	Side length of the bottom hexagon	15 mm
b	Length of the mountain crease	20.81 mm
α	Angle between the mountain and valley creases	30 degree

Table S2. Printing parameters in Ideamaker

Parameter	Value	Unit
**Layer Settings**		
Layer Height	0.1	mm
First Layer Height	0.2	mm
**Extruder Settings**		
Left Extrusion (TPE) Width	0.42	mm
Right Extrusion (PLA) Width	0.4	mm
Retraction Speed	5.0	mm/s
Retraction Material Amount	0.5	mm
**Cooling Settings**		
Fan Speed	100	%
**Tempertature Settings**		
Heated Bed Temperature	60	°C
Left Extruder	230	°C
Right Extruder	215	°C
**Speed Settings**		
Default Printing Speed	10.0	mm/s
First Layer Speed	5.0	mm/s
Support Speed	15.0	mm/s
Thin Wall Speed	7.5	mm/s
**Infill Settings**		
Infill Density	50	%
Infill Flowrate	100	%
Infill Pattern Type	Grid	-
**Support Settings**		
Generate Support	All	-
Support Extruder	Left Extruder	-
Max Overhang Angle	45	Deg
Horizontal Offset	0.50	mm
**Platform Additions Settings**		
Platform Addition	NONE	-
**Other Settings**		
Force Retraction ON Layer Change	ENABLED	-
Avoid Retraction at Start	ENABLED	-
Wipe Wall	DISABLED	-
Wipe Tower	DISABLED	-

Table S3. Hyper-elastic material model constants

Material Constant	Value
C_{10}	0.681 MPa
C_{01}	1.128 MPa
Incompressibility Parameter D_1	0.000 MPa^{-1}

Table S4. Symbol explanation in algorithm 1

Symbol	Description
$\boldsymbol{L}$	Prescribed trajectory
$\boldsymbol{P}_1^1$	Initial position of the forefoot
$\boldsymbol{P}_2^1$	Initial position of the hindfoot
$\boldsymbol{P}_{\text{final}}$	Endpoint of $\boldsymbol{L}$
e	Distance between the forefoot and $\boldsymbol{P}_{\text{final}}$
$\boldsymbol{P}_1^i$	Position of the forefoot before step i
$\boldsymbol{P}_2^i$	Position of the hindfoot before step i
$\boldsymbol{P}_1^{i+1}$	Position of the forefoot after step i
$\boldsymbol{P}_2^{i+1}$	Position of the hindfoot after step i
$\mathcal{F}_1$	Gait Controller, aka Algorithm S1

Table S5. Symbol explanation in algorithm S1

Symbol	Description
d	Forward-looking distance
$\mathcal{P}^i_{\text{goal}}$	Set of available forefoot-placement points in step i
$\boldsymbol{P}^i_{\text{goal}}$	Goal position of the pure pursuit controller in step i
$\boldsymbol{z}'^i$	Orientation of SPARC
$\boldsymbol{L}^i_{pp}$	Tracking path generated by the pure pursuit controller
$\mathcal{F}_2$	Pure pursuit Controller, aka Algorithm S2
$\hat{\boldsymbol{P}}^{i+1}_1$	Estimated landing point of the forefoot in step i
$\boldsymbol{l}^{i+1}$	Lengths of actuators, including $\{l^{i+1}_j, j = 1, 2, 3\}$
$\boldsymbol{l}^{\hat{i+1}}$	Temporary variable for storing data $\boldsymbol{l}^{i+1}$.
$\boldsymbol{\phi}^{i+1}$	Twisting angles of actuators, including $\{\phi^{i+1}_j, , j = 1, 2, 3\}$
$\mathcal{F}_3$	Inverse kinematic model, aka Algorithm S3
$\Delta\phi_{\text{current}}$	Incremental angles solved by the Quasi-Newton method
$f()$	Self-sensing model, aka Eq. (S5)
$f'()$	Derivative function of f

Table S6. Symbol explanation in algorithm S2 and S4

Symbol	Description
$\boldsymbol{P}_o^i$	Center of the circular arc $\boldsymbol{L}_{pp}^i$
R^i	Radius of the circular arc $\boldsymbol{L}_{pp}^i$
Ω^i	Central angle of the circular arc $\boldsymbol{L}_{pp}^i$
$\boldsymbol{\Phi}^{i+1}$	Feedback angles through the sensors
K_p	Proportional Gain
K_i	Integral Gain
K_d	Derivative Gain
$\boldsymbol{e}^{i+1}$	Error between calculated and sensed angles
$\boldsymbol{P}^{i+1}$	Proportional Term
$\boldsymbol{I}^{i+1}$	Integral Term
$\boldsymbol{D}^{i+1}$	Derivative Term
$\boldsymbol{u}^{i+1}$	Control Signal

Table S7. Parameters in proprioception model

Symbol	Quantity	Value
n	Number of sections	6
r	Length of the base hexagon's side	14 mm
A_0	Parameter obtained from data fitting	9.6081
B_0	Parameter obtained from data fitting	0.0160
C_0	Parameter obtained from data fitting	7.9928

Table S8. Wall thickness optimization results for the Kresling origami actuator. (The parameter values used in this work are highlighted in red.)

Thickness (mm)	Maximum stress at -60 kPa (Mpa)
0.4	31.44
0.5	10.19
0.6	5.57
0.7	3.51
0.8	2.95
0.9	2.11

Table S9. Optimization results for the Kresling origami actuator parameters h_0 and b (The parameters used in this work are highlighted in red.)

h_0 (mm)	b (mm)	Contraction ratio at -60 kPa (%)
15	16.71	42.25
16	17.54	48.64
17	18.37	52.10
18	19.19	53.85
19	20.00	54.78
20	20.81	57.12
21	21.63	60.34
22	22.45	52.59
23	23.29	38.49
24	24.15	18.59
25	25.04	8.52

Algorithm S1 Gait controller of step i, namely $\mathcal{F}_1$

Input: $\boldsymbol{L}, \boldsymbol{P}_1^i, \boldsymbol{P}_2^i$.
Output: $\boldsymbol{P}_1^{i+1}, \boldsymbol{P}_2^{i+1}$.

Initialize d
$\boldsymbol{P}_{\text{final}} \leftarrow$ Endpoint of $\boldsymbol{L}$
$\mathcal{P}_{\text{goal}}^i \leftarrow$ Solve $\left\|\overrightarrow{\boldsymbol{P}_1^i \boldsymbol{P}_{\text{goal}}^i}\right\| \leq d \wedge \boldsymbol{P}_{\text{goal}}^i \in \boldsymbol{L}$
$\boldsymbol{P}_{\text{goal}}^i \leftarrow \min(\left\|\overrightarrow{\boldsymbol{P}_{\text{final}} \boldsymbol{P}_{\text{goal}}^i}\right\|) \wedge \boldsymbol{P}_{\text{goal}}^i \in \mathcal{P}_{\text{goal}}^i$.
$\boldsymbol{z}'^i \leftarrow \overrightarrow{\boldsymbol{P}_2^i \boldsymbol{P}_1^i}$.
$\boldsymbol{L}_{pp}^i \leftarrow \mathcal{F}_2(\boldsymbol{P}_1^i, \boldsymbol{P}_{goal}^i, \boldsymbol{z}'^i)$.
for $\hat{\boldsymbol{P}}_1^{i+1} = \boldsymbol{P}_1^i$ to $\boldsymbol{P}_{\text{goal}}^i$ and $\hat{\boldsymbol{P}}_1^{i+1} \in \boldsymbol{L}_{pp}^i$ **do**
    $\hat{\boldsymbol{l}}^{i+1} \leftarrow \mathcal{F}_3(\hat{\boldsymbol{P}}_1^{i+1})$.
    if $\hat{l}_j^{i+1} < l_{\text{min}} \vee \hat{l}_j^{i+1} > l_{\text{max}}, j = 1, 2, 3$ **then**
        Break
    end if
    $\boldsymbol{l}^{i+1} \leftarrow \hat{\boldsymbol{l}}^{i+1}$
end for
Initialize $\boldsymbol{\phi}^{i+1}$.
for $j = 1$ to 3 **do**
    $\Delta\phi_{\text{current}} \leftarrow \frac{f(\phi_j^{i+1}) - l_j^{i+1}}{f'(\phi_j^{i+1})}$.
    while $\|\Delta\phi_{\text{current}}\| \geq \varepsilon$ (the precision criterion) **do**
        $\phi_j^{i+1} \leftarrow \phi_j^{i+1} - \Delta\phi_{\text{current}}$
        $\Delta\phi_{\text{current}} \leftarrow \frac{f(\phi_j^{i+1}) - l_j^{i+1}}{f'(\phi_j^{i+1})}$.
    end while
end for
Fix the hindfoot and **release** the forefoot.
PID controller $\mathcal{F}_4(\boldsymbol{\phi}^{i+1})$
Fix the forefoot and **release** the hindfoot.
Contract the body to the shortest.
$\boldsymbol{P}_1^{i+1}, \boldsymbol{P}_2^{i+1} \leftarrow$ Gain the position of fore and hindfoot.
Return $\boldsymbol{P}_1^{i+1}, \boldsymbol{P}_2^{i+1}$

Algorithm S2 Pure pursuit Controller, namely $\mathcal{F}_2$

Input: $\boldsymbol{P}_1^i, \boldsymbol{P}_{\text{goal}}, \boldsymbol{z}'^i$.
Output: $\boldsymbol{L}_{pp}^i$ including $\boldsymbol{P}_o^i, R^i, \Omega^i$.

$\boldsymbol{P}_o^i, R^i, \Omega^i \leftarrow$ Solve equations (4) using $\boldsymbol{P}_1^i, \boldsymbol{P}_{\text{goal}}^i, \boldsymbol{z}'^i$.
$\boldsymbol{L}_{pp}^i \leftarrow \boldsymbol{P}_o^i, R^i, \Omega^i$
Return $\boldsymbol{L}_{pp}^i$

Algorithm S3 **Inverse kinematic model, namely** $\mathcal{F}_3$

Input: $\boldsymbol{P}_1^{i+1}$.

Output: $\boldsymbol{l}_{i+1}$.

$(x, y, z) \leftarrow \boldsymbol{P}_1^{i+1}$

Calculate (l_1, l_2, l_3) using equation (2) and (x, y, z)

$\boldsymbol{l}_{i+1} \leftarrow (l_1, l_2, l_3)$

Return $\boldsymbol{l}_{i+1}$

Algorithm S4 **PID Controller, namely** $\mathcal{F}_4$

Procedure ($\boldsymbol{\phi}^{i+1}$).

Initialize K_p, K_i, K_d

Gain the value of sensors $\boldsymbol{\Phi}^{i+1}$

Initialize error $\boldsymbol{e}^{i+1} \leftarrow \boldsymbol{\phi}^{i+1} - \boldsymbol{\Phi}^{i+1}$

for $j = 1$ to M **do** (the prescribed times)

    $\boldsymbol{P}^{i+1} \leftarrow K_p \cdot \boldsymbol{e}^{i+1}$

    $\boldsymbol{I}^{i+1} \leftarrow K_i \cdot \sum \boldsymbol{e}^{i+1}$

    $\boldsymbol{D}^{i+1} \leftarrow K_d \cdot \frac{d\boldsymbol{e}^{i+1}}{dt}$

    Compute control signal: $\boldsymbol{u}^{i+1} \leftarrow \boldsymbol{P}^{i+1} + \boldsymbol{I}^{i+1} + \boldsymbol{D}^{i+1}$

    Apply $\boldsymbol{u}^{i+1}$ to the system

    Update $\boldsymbol{\Phi}^{i+1}$

    $\boldsymbol{e}^{i+1} \leftarrow \boldsymbol{\phi}^{i+1} - \boldsymbol{\Phi}^{i+1}$

end for

Supplementary Movies

Movie S1. Proprioception Modeling of the Kresling Origami Actuator
Movie S2. S-shape Curve Trajectory Following with Real-time Reconstruction
Movie S3. S-shape Square Trajectory Following with Real-time Reconstruction
Movie S4. Vertical Curved Trajectory Path Following with Real-time Reconstruction
Movie S5. Vertical Straight Line Following (500 g) with Real-time Reconstruction
Movie S6. Ground-to-Wall Transition
